# Variation in Plastome Sizes Accompanied by Evolutionary History in Monogenomic Triticeae (Poaceae: Triticeae)

**DOI:** 10.3389/fpls.2021.741063

**Published:** 2021-12-13

**Authors:** Ning Chen, Li-Na Sha, Yi-Ling Wang, Ling-Juan Yin, Yue Zhang, Yi Wang, Dan-Dan Wu, Hou-Yang Kang, Hai-Qin Zhang, Yong-Hong Zhou, Gen-Lou Sun, Xing Fan

**Affiliations:** ^1^Triticeae Research Institute, Sichuan Agricultural University, Chengdu, China; ^2^College of Life Science, Shanxi Normal University, Shanxi, China; ^3^Lijiang Nationality Secondary Specialized School, Lijiang, China; ^4^Saint Mary’s University, Halifax, NS, Canada

**Keywords:** chloroplast genome, Triticeae, genome variation, genome size, IR expansion/contraction

## Abstract

To investigate the pattern of chloroplast genome variation in Triticeae, we comprehensively analyzed the indels in protein-coding genes and intergenic sequence, gene loss/pseudonization, intron variation, expansion/contraction in inverted repeat regions, and the relationship between sequence characteristics and chloroplast genome size in 34 monogenomic Triticeae plants. Ancestral genome reconstruction suggests that major length variations occurred in four-stem branches of monogenomic Triticeae followed by independent changes in each genus. It was shown that the chloroplast genome sizes of monogenomic Triticeae were highly variable. The chloroplast genome of *Pseudoroegneria*, *Dasypyrum*, *Lophopyrum*, *Thinopyrum*, *Eremopyrum*, *Agropyron*, *Australopyrum*, and *Henradia* in Triticeae had evolved toward size reduction largely because of pseudogenes elimination events and length deletion fragments in intergenic. The *Aegilops*/*Triticum* complex, *Taeniatherum*, *Secale*, *Crithopsis*, *Herteranthelium*, and *Hordeum* in Triticeae had a larger chloroplast genome size. The large size variation in major lineages and their subclades are most likely consequences of adaptive processes since these variations were significantly correlated with divergence time and historical climatic changes. We also found that several intergenic regions, such as *pet*N–*trn*C and *psb*E–*pet*L containing unique genetic information, which can be used as important tools to identify the maternal relationship among Triticeae species. Our results contribute to the novel knowledge of plastid genome evolution in Triticeae.

## Introduction

Chloroplast DNA (cp DNA) is composed of a single circular DNA molecule with a quadripartite structure and encodes multiple proteins, including components of light reactions in the photosynthesis process (Martin et al., 2002). In angiosperms, cp genomes have a very constrained size, ranging from 115 to 165 kb in length, and consist of two copies of an inverted repeat (IR) region, a large-single-copy (LSC) region, and a small single copy (SSC) region (Raubeson and Jansen, 2005; Wicke et al., 2011). Because of a uniparental mode of inheritance and high conservation in gene content and genome structure, the chloroplast genome is generally treated as a single locus (Raubeson and Jansen, 2005; Nock et al., 2011). With a smaller effective population size and being essentially recombination-free, the chloroplast genome has a shorter coalescent time than nuclear genomes (Birky et al., 1983). The cp genome is highly conserved in structure, size, and gene content within land plants (Palmer, 1985; Wicke et al., 2011), because chloroplast sequences evolve at approximately half the speed of nuclear regions (Walker et al., 2014). However, variations in some regions of the cp genome have been widely reported in plants (Krause, 2011; Weng et al., 2014; Schwarz et al., 2015; Chen et al., 2017; Bedoya et al., 2019; Shrestha et al., 2019). These advantageous features give the chloroplast genome an important value in reconstructing phylogeny, DNA barcoding for accurate identification of plant species, and tracing evolutionary history (Jansen et al., 2008; Walker et al., 2014; Chen et al., 2017; Bedoya et al., 2019; Shrestha et al., 2019). However, associations between DNA composition and cp genome divergence need to be clarified in species over a range of evolutionary time.

Comparison of whole cp genomes can explore sequence variation, permit examination of molecular evolutionary patterns associated with structural rearrangement, and elucidate genetic changes underlying those events. Previous studies on seed plants have suggested that structural rearrangement, intergenic region variation, variation in IR regions, and gene loss were principal factors driving the variation in chloroplast genome size and structure (Kugita et al., 2003; Parks et al., 2009; Wu et al., 2011; Chen et al., 2017; Zheng et al., 2017). It has been reported that contractions into single copy regions with inversions have shaped certain evolutionary features for monophyletic groups (Palmer et al., 1985; Hoot and Palmer, 1994). While these described features in chloroplast genome variation enable researchers to investigate genome divergences over a broad range of evolutionary time, from early land plants to recently domesticated plants, comparisons among very distant relatives have yielded results with uncertain generality (Matsuoka et al., 2002; Zheng et al., 2017). Moreover, a comprehensive search for factors that drive the variation in genome size in a given phylogenetic framework is still lacking.

The wheat tribe (*Poaceae*: Triticeae), an economically important gene pool for genetic improvement of cereal and forage crops, includes about 450 diploid and polyploid species that distribute in a wide range of ecological habitats over temperate, subtropical, and tropic alpine regions (Dewey, 1984). In Triticeae, 24 major basic genomes in diploid species have been designated and recognized in 18 monogenomic genera (Löve, 1984; Wang et al., 1994). Monogenomic genera have been the focus of numerous evolutionary investigations, partly because of either their economic importance (including barley, rye, and crested-wheat grasses) or their genome donor to the speciation of polyploid species (accounting for 75% of the Triticeae species), as well as their wide variety of species richness, morphology, ecology, and distribution. Previous data from geographical distribution (Sakamoto, 1973) and morphological characteristics (West et al., 1988), and DNA information (Kellogg et al., 1996) have suggested that the first step of phylogenetic diversification in Triticeae occurred at the diploid level and then gave rise to different present-day diploid lineages. Since the tribe was originated, lineage diversification of monogenomic genus in a range of evolutionary time raised the question of how cp genomes have evolved. Comparison of cp genomic structure on several monogenomic Triticeae showed that a 737-bp deletion in *Pseudoroegneria libanotica* might be related to potential nuclear–cytoplasm transfer (Wu et al., 2017). Analysis of the cp genome sequence in *Agropyron cristatum* suggested that deletion of *acc*D and translocation of *rpl*23 genes might represent an independent gene-loss event or an additional divergence in Triticeae (Chen et al., 2017). However, to better understand the pattern of variation in cp genome structure and size in Triticeae, it is critical to assess the evolutionary processes that drive cp genome variation accompanied by the diversification of Triticeae with relatively well-sampled monogenomic Triticeae genera.

Here, we carried out phylogenetic reconstructions and estimated diversification patterns using the cp genome from 34 species of 17 monogenomic genera within the Triticeae. While generic definitions within the Triticeae have been extremely variable (Barkworth, 1998), our sample represents nearly all monogenomic genera and genome types according to genome-based classifications of the tribe (Yen et al., 2005). The aims of this study were to (1) examine variation in cp genomic structure and size in monogenomic Triticeae; (2) document the process of cp genomic variation accompanied by the diversification history of monogenomic Triticeae. Knowledge of cp genomic variation over a range of evolutionary time would provide a better understanding of the evolutionary history of the Triticeae.

## Materials and Methods

### Data Collection

Thirty-five species, which included 34 individuals representing 17 genera and 20 basic genomes within Triticeae, were sampled (Table 1). *Brachypodium distachyon* was included as an outgroup. Twenty chloroplast genome sequences are highlighted in bold in Table 1, which are from *Triticum–Aegilops* complex, *Hordeum bulbosum*, *Hordeum vulgare*, *Hordeum vulgare* ssp. *spontaneum*, *Secale cereale*, were downloaded from the NCBI published data (Saski et al., 2006; Gogniashvili et al., 2014, NCBI web, Bortiri et al., 2008; Gornicki et al., 2014; Middleton et al., 2014; Saarela et al., 2015). The remaining sequences were from our previous publications (Chen et al., 2017, 2020; Wu et al., 2017).

**TABLE 1:** List of taxa used in this study.

No.	Sequence number	Species	Accession no.	Genome	Ploidy	Origin	GeneBank	Chloroplast genome size (bp)
1	KJ614418	*Aegilops bicornis*	Clae57	S^b^	2×	SE Mediterranean	KJ614418	136,861
2	KJ614416	*Aegilops longissima*	TA1924	S^l^	2×	E Mediterranean	KJ614416	136,875
3	KY636033	*Aegilops markgrafii*	AE 1381	C	2×	NE Mediterranean	KY636033	136,063
4	KJ614413	*Aegilops searsii*	TA1926	S^S^	2×	E Mediterranean	KJ614413	136,870
5	KJ614419	*Aegilops sharonensis*	TA1995	S^sh^	2×	Israel, Lebanon	KJ614419	136,867
6	KJ614406	*Aegilops speltoides*	PI487232	S	2×	E Mediterranean	KJ614406	135,652
7	KJ614405	*Aegilops speltoides* ssp. *ligustica*	TA1796	S	2×	E Mediterranean	KJ614405	135,660
8	KJ614412	*Aegilops tauschii*	Cultivar AL8/78	D	2×	SW–C Asia	KJ614412	135,568
10	KY636059	*Aegilops umbellulata*	AE 153	U	2×	SE Europe, SW Asia	KY636059	136,028
9	KY636056	*Aegilops umbellulata* ssp. *transcaucasica*	AE 1070	U	2×	SE Europe, SW Asia	KY636056	136,031
11	C22	*Agropyron cristatum*	PI 598628	P	2×	Kazakhstan	KY126307	135,554
12	K30	*Agropyron mongolicum*	PI 499392	P	2×	Nei Monggol, China	MH285848	135,547
13	KY636075	*Amblyopyrum muticum*	PI 560125	T	2×	Turkey	KY636075	135,787
14	E48	*Australopyrum retrofractum*	PI 533013	W	2×	NEW South Wales, Australia	MH331642	135,417
15	CRK28	*Crithopsis delileana*	ND	K	2×	Greece	MH285849	136,436
16	DAK25	*Dasypyrum villosum*	W6 7264	V	2×	Greece	MH285850	135,249
17	ERK39	*Eremopyrum distans*	TA 2229	F	2×	Afghanistan	MH285851	135,589
18	ERK31	*Eremopyrum tririceum*	Y206	Xe	2×	Xinjiang, China	MH285852	135,554
19	HEK26	*Henradia persica*	–	O	2×	Iran	MH285853	135,659
20	HEK24	*Heteranthelium piliferum*	PI401352	Q	2×	Iran	MH285854	136,768
21	E46	*Hordeum bogdanii*	PI 531761	H	2×	Xinjiang, China	MH331641	136,968
22	KM974741	*Hordeum jubatum*	CAN:Saarela 18478	H	3×	–	KM974741	136,826
23	EF115541	*Hordeum vulgare*	Morex	I	2×	Minnesota, United States	EF115541	136,462
24	KC912689	*Hordeum vulgare* ssp. *spontaneum*	FT462	I	2×	Minnesota, United States	KC912689	136,043
25	K27	*Lophopyrum elongatum*	PI531718	E^e^	2×	Tunisia	MH331643	135,020
26	C26	*Psathyrostachys juncea*	PI 430871	Ns	2×	Former soviet union	MH331640	136,597
27	E47	*Pseudoroegneria libanotica*	PI 228392	St	2×	Iran	KX822019	135,026
28	PSK29	*Pseudoroegneria spicata*	PI 232134	St	2×	Wyoming, United States	MH285855	135,165
29	KC912691	*Secale cereale*	Imperial	R	2×	Turkey	KC912691	135,564
30	TAK23	*Taeniatherum caput-medusae*	PI220591	Ta	2×	Afghanistan	MH285856	136,861
31	THC25	*Thinopyrum bessarabicum*	PI 531712	E^b^	2×	Estonia	MH331639	135,003
32	LC005977	*Triticum monococcum*	–	A	2×	Turkey	LC005977	136,886
33	KC912692	*Triticum monococcum* ssp. *aegilopoides*	–	A	2×	Turkey	KC912692	136,870
34	KJ614411	*Triticum urartu*	PI428335	A	2×	E Mediterranean, Caucasus	KJ614411	136,865
35	EU325680	*Brachypodium distachyon*	Cultivar Bd21	–	–	–	EU325680	135,199

*The GenBank accession number with bold represent previously published sequences from the GenBank (http://www.ncbi.nlm.nih.gov). ND: not determined.*

### Comparison of 35 Chloroplast Genomes

The software mVISTA was used to compare the cp genomes using the annotation of *A. cristatum* as a reference (Frazer et al., 2004). The sequences were aligned with MAFFT v6.833 (Katoh and Toh, 2010) using default settings. Sequence variation, such as gene loss, divergent genes, intergenic sequence (IGS) indels, and IR contraction/expansion, were examined by MEGA 6.0 (Tamura et al., 2013). The IR expansion and contraction of cp genomes were analyzed using IRscope (Amiryousefi et al., 2018).

### Phylogenetic Analysis

Phylogenetic analysis was performed using maximum likelihood (ML) and Bayesian inference (BI). ML analysis was conducted using RAxML v.8.2.104 (Stamatakis, 2014). The best model was determined by the model test-ng-0.1.6 software with a default parameter (Kozlov et al., 2019). The optimal model identified was GTR + I + G, which was used in both the ML and BI analyses. The robustness of the trees was estimated by bootstrap support (BS). Statistical support for nodes in ML analysis was estimated using 1,000 fast bootstrap replicates, each with three replicates of stepwise random taxon addition. A BS value of less than 50% was not included in the figures. The BI analysis was performed using MrBayes v3.0 (Huelsenbeck and Ronquist, 2001). Four Markov Chain Monte Carlo (MCMC) chains (one cold and three heated), with MrBayes default heating values (*t* = 0.2), were run for 100,000 generations, each sampling every 100 generations. The first 250 trees were stationarily discarded as “burn-in.” The statistical confidence in nodes was estimated by posterior probabilities (PP). A PP value of less than 0.9 was not included in the figures.

### Ancestral State Reconstruction

Two data matrixes, complete cp genome sequence data and concatenated non-protein coding data, were used to trace the ancestral states of genome size on the phylogenetic tree using weighted squared-change parsimony in the software Mesquite v2.5 (Maddison and Maddison, 2021). Weighted squared-change parsimony minimizes the sum of squared change along all branches of the tree, weighting branches by their length (Finarelli and Flynn, 2006). The evolutionary history of the two selected data characters was mapped over the single best ML tree based on complete cp genome sequences.

### Divergence Dating

Divergence times with 95% confidence intervals were estimated using the Bayesian relaxed molecular clock method, implemented in BEAST v1.4.6 (Drummond and Rambaut, 2007). Calibration points were performed using a relaxed uncorrelated lognormal molecular clock. A complete cp genome sequence dataset was used to conduct the BEAST analysis. The lack of fossils for Triticeae precluded the direct calibration of tree topologies. Instead, dating was based on the divergence time for the basal-most split in Triticeae (Marcussen et al., 2014). Priors on Triticeae crown age (15.32 Ma ± 0.34) were set as inferred by Marcussen et al. (2014), where several macrofossils from grass (Festuca, Berriochloa, and Nassella) were used to calibrate the age of Triticeae. Tracer v1.4 (Rambaut and Drummond, 2018) was used to ensure the convergence of mixing in terms of effective sample size (ESS) values and coefficient rate. The results will be accepted if the values of the estimated sample size were larger than 200, suggesting little autocorrelation between samples. The resultant trees were analyzed using TreeAnnotator in BEAST where the “burn-in” (2,000 trees) was removed, and a maximum credibility tree was constructed. The trees were then viewed in FigTree v. 1.3.1.^1^

### Statistical Analysis

Statistical analysis was performed by using the R software. Data statistics of the complete cp genome, protein coding region, and non-protein coding region of each clade were compared with Boxplot. Kruskal–Wallis test was performed on the complete cp genome, protein coding region, and non-protein coding region to determine significant differences among the clades. The relationship between divergence time and indel amount was evaluated by using Spearman’s correlation coefficient test.

## Results

### Phylogeny of the Triticeae Species

**FIGURE 1:**
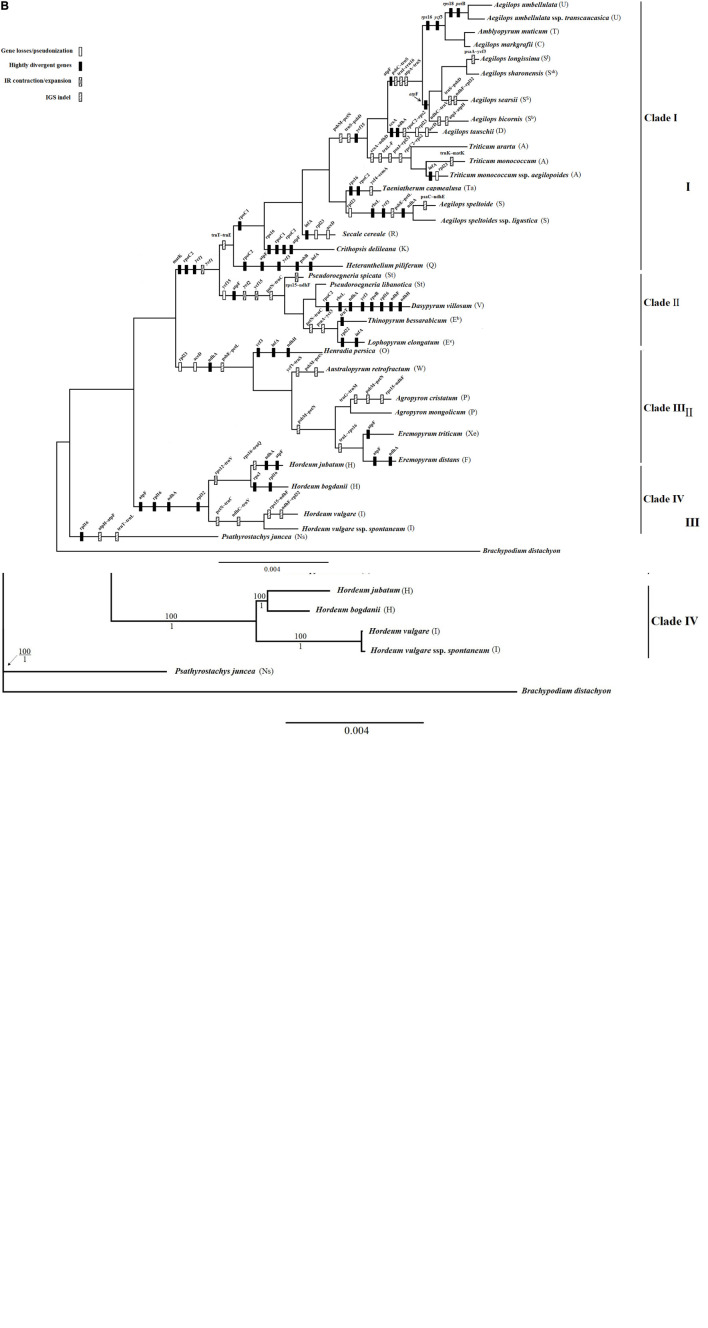
Maximum-likelihood tree inferred from whole complete chloroplast (cp) genome sequences for the diploid Triticeae using RAxML v.8.2.10. **(A)** Phylogenetic tree topology with bootstrap support (BS) above and posterior probabilities (PP) below branches (>50% BS; >0.9 PP). **(B)** Gene loss/pseudonization, indels in protein coding genes, intron variation, and intergenic sequence (IGS) indels within the cp genomes were characterized and mapped on the branches of the phylogenetic tree.

Chloroplast genomes contain an abundance of phylogenetic information, which has been widely used for phylogeny reconstruction at different taxonomic levels, such as order, family, genus, and species, in plants. Using chloroplast genome data, long-standing controversies related to various phylogenetically difficult groups have been resolved, supporting its importance in systematic studies. To better determine the phylogenetic position of Triticeae and further clarify the evolutionary relationships within the Triticeae tribe, phylogenetic analyses were constructed based on the 34 complete chloroplast genomes using *B. distachyon* as an outgroup. Phylogenetic reconstruction based on complete cp genome data resulted in a tree with high posterior probability support across most clades (Figure 1A). The ML and Bayesian analyses of complete cp genome data generated the same tree topology with BS >50% above and PP >0.9 below branches (Figure 1A). The tree illustrated that four clades (I–IV) were recognized. Clade I included the *Aegilops*/*Triticum* complex, *Taeniatherum* (Ta), *Secale* (R), *Crithopsis* (K), and *Herteranthelium* (Q), all of which are members of Mediterranean Triticeae. Clade II contained *Pseudoroegneria* (St), *Dasypyrum* (V), *Lophopyrum* (E^e^), and *Thinopyrum* (E^b^). Clade III consisted of *Eremopyrum* (F and Xe), *Agropyron* (P), *Australopyrum* (W), and *Henradia* (O). Clade IV comprised the *Hordeum* (H and I) species.

### General Variation of Chloroplast Genomes in Triticeae Species

The complete genome size of 34 cp genomes in Triticeae ranged from 135,003 (*Thinopyrum bessarabicum*) to 136,968 bp (*Hordeum bogdanii*), and non-protein coding region size from 75,694 (*T. bessarabicum*) to 77,672 bp (*H. bogdanii*). The level of sequence divergence among the 34 cp genomes was compared using the mVISTA program. We found that non-protein coding regions were more highly variable than protein coding regions, and IRs had lower sequence divergence than SC regions (Supplementary Figure 1). Otherwise, large hotspot variation regions were detected in *pet*N–*rpo*B, *rbc*L–*psa*I, and *rpl*23–*ndh*B. Gene loss/pseudonization, indels in protein coding genes, intron variation, and IGS indels within the cp genomes were characterized and mapped on branches of the phylogenetic tree (Figure 1A) of the Triticeae species based on complete plastid genomes (Figure 1B). One of the mutation events occurred between the *rbc*L gene and the *psa*I gene in the LSC region (Supplementary Figure 2). This region primarily contains the *rpl*23 gene, the *acc*D gene, and intergenic regions. The *rpl*23 and *acc*D genes were completely absent in *A. cristatum*, *Agropyron mongolicum*, *Ere. triticum*, *Ere. distant*, *Australopyrum retrofractum*, *Henradia persica*, and *Aegilops tauschii*. Within this region, the absence of a long IGS and the *acc*D gene was also detected in *Aegilops speltoides* and *Triticum monococcum* ssp. *aegilopoides* (Supplementary Figure 3). Another gene loss event was identified between the *trn*L gene and the *trn*I gene in the IRs region (Supplementary Figure 4). These deletion regions primarily contain a *ycf*2 gene fragment, the *ycf*15 gene, and intergenic regions, and occurred in *Pseudoroegneria spicata*, *P. libanotica*, *T. bessarabicum*, *Lophopyrum elongatum*, and *Dasypyrum villosum*, which were grouped in clade II (Figure 1). A hot variation region was also found in the non-protein coding regions. All species in Clade II had a 529-bp deletion between the *pet*N and *trn*C genes in LSC (Supplementary Figure 5). All species in clade III, *A. speltoides*, *Aegilops speltoides* ssp. *ligustica* and *T. monococcum* ssp. *aegilopoides*, had a 438-bp deletion between *psb*E and *pet*L in LSC (Supplementary Figure 6). All species in Clade I and *Hordeum jubatum* contained a 172-bp deletion between *trn*T and *trn*E in LSC.

### Highly Divergent Genes

The total number of protein coding genes was 76 in each cp genome of the Triticeae species. Nucleotide divergence occurred in the coding regions of *rpo*C2, *rps*3, *rpl*22, *mat*K, *ycf*1, *rps*16, *rpo*C1, *ndh*H, *atp*F, *rpl*16, *rpl*32, *ndh*A, *psb*B, *ycf*3, and *inf*A. Among these, the *rpo*C2 gene has a 6-bp insertion fragment in the species clustered in clades I and II, and a 42-bp insertion fragment in *D. villosum* (Supplementary Figures 7, 8). The *Rps*3 gene in *H. bogdanii* has a 45-bp deletion (Supplementary Figure 9). The *Inf*A gene had a deletion of two different 18-bp fragments in *L. elongatum*, *S. cereale*, and *H. jubatum* (Supplementary Figures 10, 11). A 15-bp deletion in the *rpl*22 gene was found in *L. elongatum* (Supplementary Figure 12).

### Inverted Repeat Contraction/Expansion

**FIGURE 2:**
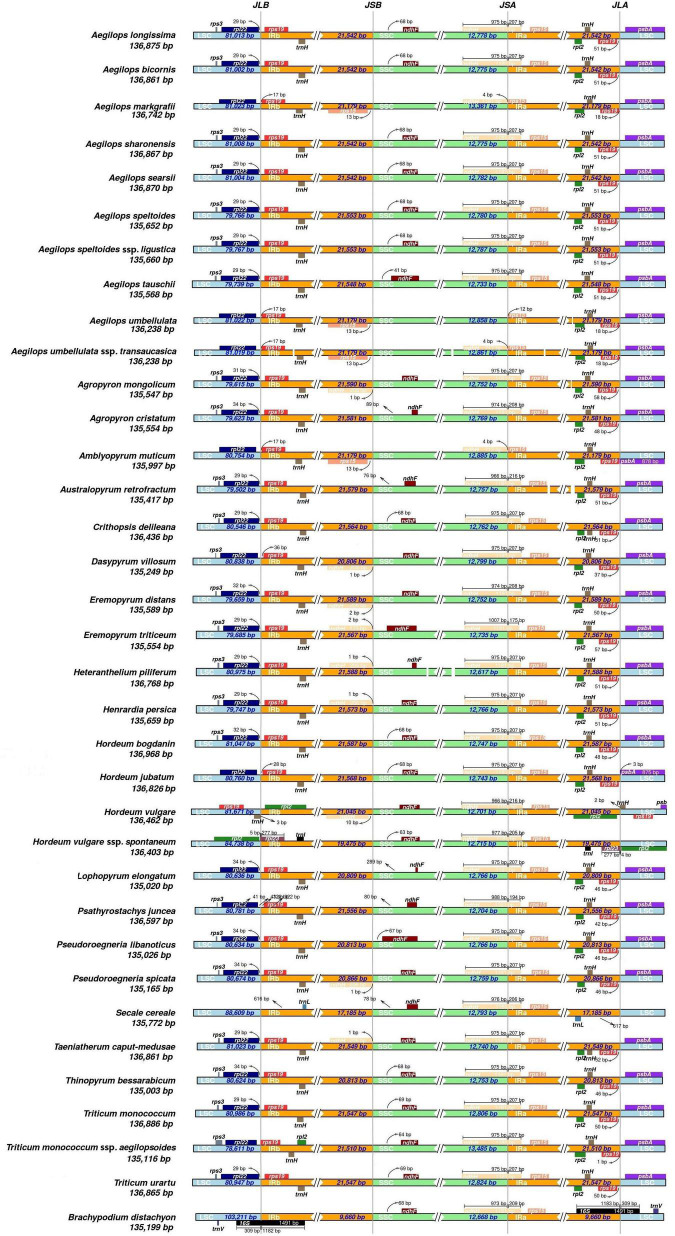
Comparison of the borders of the large-single-copy (LSC) (blue), small single copy (SSC) (green), and inverted repeat (IR) (orange) regions among the 34 cp genomes.

The most common events underlying changes in the plastome size of land plants included the contraction/expansion of IR. Land plants have a highly conserved chloroplast genome, but four junctions (LSC/IRB/SSC/IRA) vary in genome size and could affect IR contraction and expansion (Saski et al., 2007; Choi and Park, 2015). All IR boundaries among the sampled monogenomic Triticeae species were in a similar location, but IR size varied from 20,806 (*D. villosum*) to 21,589 bp (*Eremopyrum distans*) because of variation in the size of pseudogenes *ycf*2 and *ycf*15. Because of a ∼800-bp deletion of *ycf*2 and *ycf*15 in the clade II species, IR size varied from 20,806 (*D. villosum*) to 20,866 bp (*P. spicata*), which was much smaller than that in the other Triticeae species (>21 kb) (Figure 2).

The LSC/IRb/SSC/IRa boundary regions were compared using IRscope (Figure 2). The LSC/IRb junction was between the *rpl*22 gene and the *rps*19 gene in 31 Triticeae species, and close to the *rps*19 gene in the plastomes of *Aegilops markgrafii*, *Aegilops umbellulata*, *Aegilops umbellulata* ssp. *transcaucasica*, *Amblyopyrum muticum*, *D. villosum*, *H. jubatum*, and *T. monococcum* ssp. *aegilopoides*, varying from 17 to 36 bp. The *rpl*22 gene in the LSC region was close to the LSC/IRb junction in 24 out of the 31 cp genomes and varied from 28 to 42 bp apart from the LSC/IRb junction. The *Rpl*2 gene in *H. vulgare* was in IR regions 54 bp away from the LSC/IRb junction, while the *rpl*2 gene in *H. vulgare* ssp. *spontaneum* was in the LSC region 5 bp away from the LSC/IRb junction. The distance between *rps*15 and the junction of the IRb/SSC region ranged from 13 to 480 bp (except *rps*15 in *H. vulgare* that crossed the junction of the IRb/SSC region). At the junction of the SSC/IRa region, the *ndh*H gene crossed the SSC/IRa boundary in all the genomes with 0–1,007 bp located in the SSC region. The *rps*19 gene was in the IRa region in 31 of the species 1–51 bp apart from the LSC/IRa junction. The *rpl*2 gene was located in the IRa region 590 bp apart from the LSC/IRa junction in *H. vulgare*, and in the LSC region 4 bp apart from the junction in *H. vulgare* ssp. *spontaneum*.

### Ancestral State Reconstruction

**FIGURE 3:**
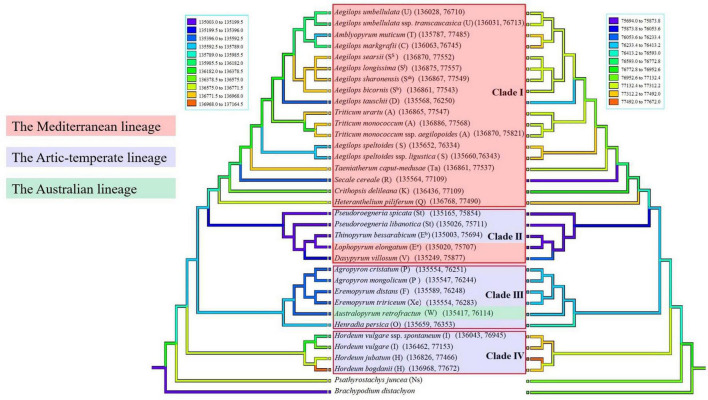
Ancestral state reconstructions were traced on the ML tree inferred from two selected data (cp genome sequences and non-protein gene sequences) using weighted squared-change parsimony. Different colors labeled the geographic information of monogenomic genera. The capital letters in the bracket indicate the genome type of the species.

Sizes of complete chloroplast genomes, protein coding sequences, and non-protein coding sequences in monogenomic Triticeae are shown in Table 2. The reconstruction of character evolution revealed that the sizes of complete cp genome sequences and non-protein coding sequences of the species within clades I and IV were gradually enlarged (Figure 3). The sizes of complete genome sequences ranged from 135,564 (*S. cereale*) to 136,886 bp (*Triticum monococcum*), and from 136,043 (*H. vulgare* ssp. *spontaneum*) to 136,968 bp (*H. bogdanii*) for the species in clades I and IV, respectively, while the sizes of complete cp genome sequences of species within clades II and III were significantly smaller than those within clades I and IV, and ranged from 135,003 bp (*T. bessarabicum*) to 135,249 bp (*D. villosum*) in clade II, and from 135,417 (*A. retrofractum*) to 135,659 bp (*H. persica*) in clade III. The sizes of protein coding sequences varied from 58,302 (*A. muticum*) to 61,049 bp (*T. monococcum* ssp. *aegilopoides*); from 59,098 (*H. vulgare* ssp. *spontaneum*) to 59,360 bp (*H. jubatum*); from 59,309 (*T. bessarabicum*) to 59,372 bp (*D. villosum*); and from 59,271 (*Eremopyrum tririceum*) to 59,341 bp (*E. distans*) for the species within clades I, IV, II, and III, respectively.

**TABLE 2:** Sizes of 34 Triticeae species with complete chloroplast genomes and non-protein/protein coding sequences in four clades.

No.	Species	Chloroplast genome size (bp)	Non-coding size (bp)	Coding size (bp)	Clade
KJ614418	*Aegilops bicornis*	136,861	77,543	59,318	Clade I
KJ614416	*Aegilops longissima*	136,875	77,557	59,318	Clade I
KY636033	*Aegilops markgrafii*	136,063	76,745	59,318	Clade I
KJ614413	*Aegilops searsii*	136,870	77,552	59,318	Clade I
KJ614419	*Aegilops sharonensis*	136,867	77,549	59,318	Clade I
KJ614406	*Aegilops speltoides*	135,652	76,334	59,318	Clade I
KJ614405	*Aegilops speltoides* ssp. *ligustica*	135,660	76,343	59,317	Clade I
KJ614412	*Aegilops tauschii*	135,568	76,250	59,318	Clade I
KY636059	*Aegilops umbellulata*	136,028	76,710	59,318	Clade I
KY636056	*Aegilops umbellulata* ssp. *transcaucasica*	136,031	76,713	59,318	Clade I
KY636075	*Amblyopyrum muticum*	135,787	77,485	58,302	Clade I
KC912691	*Secale cereale*	135,564	77,109	58,455	Clade I
MH285856	*Taeniatherum caput-medusae*	136,861	77,537	59,324	Clade I
LC005977	*Triticum monococcum*	136,886	77,568	59,318	Clade I
KC912692	*Triticum monococcum* ssp. *aegilopoides*	136,870	75,821	61,049	Clade I
KJ614411	*Triticum urartu*	136,865	77,547	59,318	Clade I
MH285849	*Crithopsis delileana*	136,436	77,109	59,327	Clade I
MH285854	*Heteranthelium piliferum*	136,768	77,490	59,278	Clade I
Average		136,362	77,053	59,308	
KX822019	*Pseudoroegneria libanotica*	135,026	75,711	59,315	Clade II
MH285855	*Pseudoroegneria spicata*	135,165	75,854	59,311	Clade II
MH285850	*Dasypyrum villosum*	135,249	75,877	59,372	Clade II
MH331643	*Lophopyrum elongatum*	135,020	75,707	59,313	Clade II
MH331639	*Thinopyrum bessarabicum*	135,003	75,694	59,309	Clade II
Average		135,093	75,769	59,324	
KY126307	*Agropyron cristatum*	135,554	76,251	59,303	Clade III
MH285848	*Agropyron mongolicum*	135,547	76,244	59,303	Clade III
MH331642	*Australopyrum retrofractum*	135,417	76,114	59,303	Clade III
MH285851	*Eremopyrum distans*	135,589	76,248	59,341	Clade III
MH285852	*Eremopyrum tririceum*	135,554	76,283	59,271	Clade III
MH285853	*Henradia persica*	135,659	76,353	59,306	Clade III
Average		135,553	76,249	59,305	
MH331641	*Hordeum bogdanii*	136,968	77,672	59,296	Clade IV
KM974741	*Hordeum jubatum*	136,826	77,466	59,360	Clade IV
EF115541	*Hordeum vulgare*	136,462	77,153	59,309	Clade IV
KC912689	*Hordeum vulgare* ssp. *spontaneum*	136,043	76,945	59,098	Clade IV
Average		136,575	77,309	59,266	

### Divergence Dating

**FIGURE 4:**
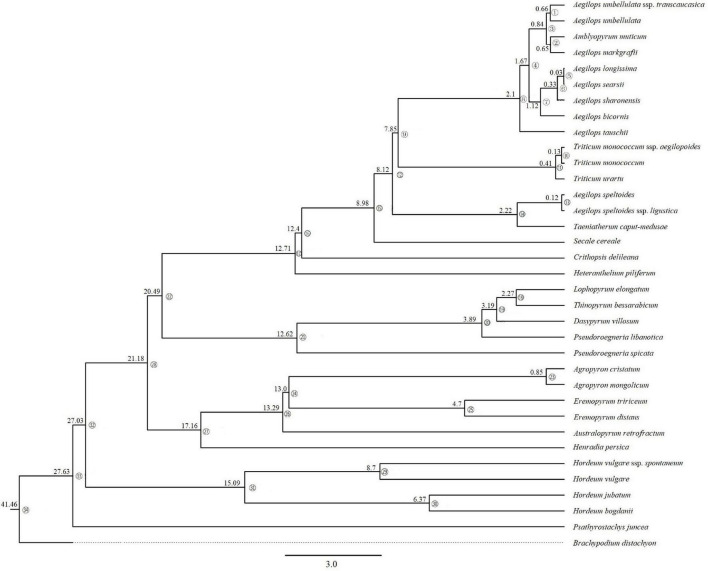
Maternal time-calibrated phylogeny was estimated based on complete cp genomes of Triticeae with 95% confidence intervals BEAST analyses.

Based on the complete cp genome sequences dataset, divergence times with 95% CI by using BEAST analyses generated a maternal time-calibrated tree (Figure 4). The divergence time was marked on 33 branch nodes within the ML tree. Time calibration analysis demonstrated that the time to the most recent common maternal ancestor of the Triticeae was dated to 27.63 MYA (95% CI). The maternal ancestor of *Hordeum* originated about 15.09 MYA (95% CI). The divergence time of maternal ancestor of species in clade III (*Agropyron*, *Australopyrum*, *Henradia*, and *Eremopyrum*) and clade II (*Dasypyrum*, *Pseudoroegneria*, *Lophopyrum*, and *Thinopyrum*) was dated to 17.16 MYA (95% CI) and 12.62 MYA (95% CI), respectively. The divergence time of the maternal ancestor of *Aegilops*/*Triticum*, *Taeniatherum*, *Secale*, *Crithopsis*, and *Heteranthelium* in clade I was 12.71 MYA (95% CI).

### Statistical Analysis

**FIGURE 5:**
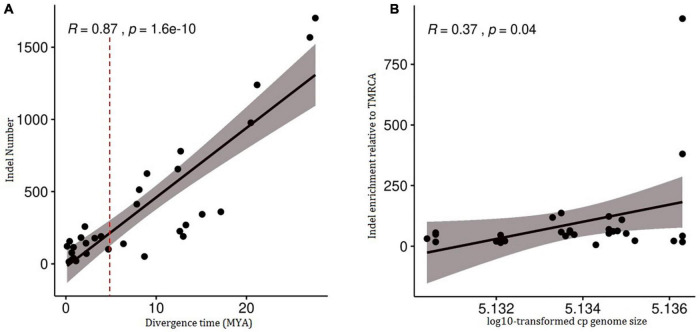
Correlation tests of number of indel in 34 chloroplast genomes of Triticeae species against divergence time and chloroplast genomes size. **(A)** A correlation test of number of indel against divergence time (*R* = 0.87, *p* = 1.6e-10<0.05). **(B)** A correlation test of number of indel against chloroplast genomes size (*R* = 0.37, *p* = 0.04).

The insertion and deletion of 33 divergence nodes of the phylogenomic tree are counted in Table 3. The divergence time was highly correlated with the number of variations (*R* = 0.87, *p* = 1.6e^–10^ < 0.05) (Figure 5A), as well as cp genome size and indel number (*R* = 0.37, *p* = 0.04) (Figure 5B). Therefore, both early and late divergence times might lead to a similar trend in the associated number of indels. The highest frequency of indels occurred about 5 MYA.

**TABLE 3:** Divergence time and indels in every node of 34 Triticeae species in the phylogenomic tree.

Node	Divergence time (MYA)	Indel	CP genome size
1	0.66	28	136,030
2	0.65	77	135,925
3	0.84	115	135,977
4	1.67	182	136,423
5	0.03	–	136,873
6	0.33	14	136,871
7	1.12	20	136,868
8	2.1	258	136,328
9	7.85	413	136,464
10	0.13	122	136,878
11	0.41	156	136,874
12	8.12	513	136,383
13	0.12	–	135,656
14	2.22	143	136,058
15	8.98	625	136,332
16	12.4	656	136,338
17	12.71	780	136,362
18	2.27	71	135,012
19	3.19	178	135,091
20	3.89	190	135,075
21	12.62	226	135,093
22	20.49	977	136,086
23	0.85	39	135,551
24	13	190	135,561
25	4.7	101	135,572
26	13.29	269	135,532
27	17.16	360	135,553
28	21.18	1,239	135,976
29	8.7	51	136,253
30	6.37	139	136,804
31	15.09	343	136,528
32	27.03	1,568	136,043
33	27.63	1,702	136,059

**FIGURE 6:**
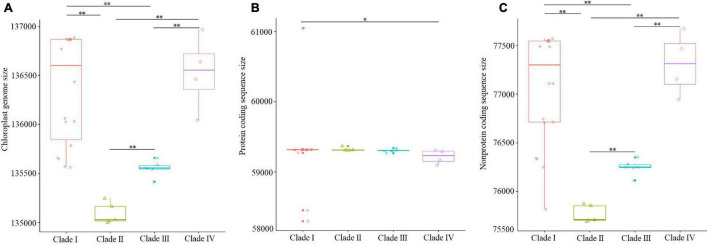
Comparisons of complete cp genome, protein coding region, and non-protein coding region within the four clades, with significant differences (*p* < 0.05, Kruskal–Wallis test) being estimated. **(A)** Comparisons of complete cp genome sequences; **(B)** comparisons of protein coding gene sequences; **(C)** comparisons of non-protein coding sequences. *p < 0.05; **p < 0.01.

The sizes of complete genome sequences and non-protein coding sequences of the species within clades I (*Triticum*/*Aegilops*) and IV (*Hordeum*) were significantly larger than those of the species within clades II (St/E/V genomes) and III (P/F/Xe/W/O genomes) (Figures 6A,C). However, the sizes of the protein coding sequences among these species were not significantly different (Figure 6B). The average sizes of complete cp genome sequences of the species within clades I–IV were 136,362, 135,093, 135,553, and 136,575 bp (Figure 6A), and protein-coding gene sequences were 59,308, 59,324, 59,305, and 59,266 bp (Figure 6B), respectively.

## Discussion

Plastome size variations within major lineages are mainly attributable to a combination of three factors: differences in length of intergenic regions, changes in intron content, and contraction/expansion of IRs, which resulted in cp genome size ranging from 115 to 165 kb in the length of angiosperms (Raubeson and Jansen, 2005; Brouard et al., 2010; Wicke et al., 2011; Smith et al., 2013; Turmel et al., 2017). Previous studies have shown that the size of the cp genome of Triticeae species ranged from 134 to 137 kb (Gornicki et al., 2014; Middleton et al., 2014; Chen et al., 2017). The intergenic regions, which represent up to 68% of the genome, contribute to most of the observed genome size variation (Turmel et al., 2015). Our ancestral genome reconstruction suggests that major rearrangements occurred in four branches (clades I–IV) of monogenomic Triticeae followed by minor independent rearrangements in each genus. The sizes of complete genome sequences of the species within clades I (*Triticum*/*Aegilops* complex, *Taeniatherum*, *Secale*, *Crithopsis*, and *Herteranthelium*) and IV (*Hordeum*) were significantly larger than those of within clades II (*Pseudoroegneria*, *Dasypyrum*, *Lophopyrum*, and *Thinopyrum*) and III (*Agropyron*, *Australopyrum*, *Eremopyrum*, and *Henradia*).

The amounts of IGSs showed extensive fluctuations and were attributed to major plastome size variation among the 34 taxa studied (Figures 3, 6), such as the 529-bp deletion between the *pet*N gene and the *trn*C gene (Supplementary Figure 5) in the species within the clade II (*Pseudoroegneria*, *Dasypyrum*, *Lophopyrum*, and *Thinopyrum*), and the 438-bp deletion between *psb*E and *pet*L in the species within clade III (*Agropyron*, *Australopyrum*, *Eremopyrum*, and *Henradia*) (Supplementary Figure 6). These deletions might occur in the ancestral plastid genomes of species in clades II and III, inherited by and maintained in their offspring species. Variations in intergenic regions do not affect protein function and structure but cause variations in chloroplast genomes’ size. Intergenic regions in the cp genome have been utilized for systematic studies in diverse plants because they provide a wealth of information for distinguishing different species, such as *trn*L–*trn*F and *trn*H–*psb*A (Sha et al., 2014; Sevindik et al., 2021). Therefore, these intergenic regions (like *pet*N–*trn*C and *psb*E–*pet*L) can be used as an important tool to identify the maternal relationship among Triticeae species in the future.

The amount of introns, such as the intron of *atp*F (Supplementary Figure 16) and the intron of *rpl*16 (Supplementary Figure 17), also showed extensive changes, in LSC, but no obvious patterns can be discerned in a phylogenetic context. The size variations in those introns were most likely the consequence of non-adaptive processes. However, random genetic drift might also play a central role in the shaping of plastome architecture (Smith, 2016).

Inverted repeat expansion and contraction were mostly associated with cp genome size variation (McCoy et al., 2008; Wu et al., 2009). Expansion and contraction in IRs of chloroplast genomes have been widely reported in various kinds of plants (Krause, 2011; Weng et al., 2014; Schwarz et al., 2015; Shrestha et al., 2019; He et al., 2020; Guo et al., 2021). We also found that cp genomes in the 34 Triticeae species exhibited obvious different IR sizes. A ∼800-bp length variation in IR was detected between *trn*I and *trn*L, which mainly contained pseudogenes *ycf*2 and *ycf*15. Wu et al. (2017) suggested that this deletion in the cp of *P. libanotica* was specific in Triticeae species. We confirmed that the loss of pseudogenes *ycf*2 and *ycf*15 occurred in the cp of *Pseudoroegneria*, *Dasypyrum*, *Lophopyrum*, and *Thinopyrum*. The *Lophopyrum*, *Thinopyrum*, and *Dasypyrum* genomes contributed a cytoplasm genome to the *Pseudoroegneria* species as a result of incomplete lineage sorting and/or chloroplast capture (Chen et al., 2020). A maternal donor of the species in clade II might have lost pseudogenes *ycf*2, *ycf*15, and adjacent gene spacers, and genetically transmitted to their offspring, leading to the IR size variation that ranged from 20,806 (*D. villosum*) to 20,866 bp (*P. spicata*), which was smaller than that in the other Triticeae species (>21 kb) (Figure 2). In some plants, *ycf*2-encoded protein and five related nuclear-encoded *Fts*H comprised a 2-MD complex, which can promote ATP synthesis (Kikuchi et al., 2018). The disappearance of *ycf*2 might indicate that this function region has been transferred to a nuclear genome. Wu et al. (2017) found that this deletion was exactly similar (identities = 99%) to a genomic scaffold of chromosome 3B of *T. aestivum*, and that it possesses high similarity (identify = 98%) with cp sequences in Pooideae species, but the specific function is still unknown. Although many species in different lineages contain an intact *ycf*15 gene (encoding the beta subunit of acetyl-CoA carboxylase complex) and have been annotated in several sequenced chloroplast genomes, e.g., several *Asterids*, *Magnolia* (Kuang et al., 2011), and *Piper* (Cai et al., 2006), it is almost impossible to determine whether this gene is able to encode a functional protein or how it has evolved in angiosperms so far. The gene was first identified in the *Nicotiana* chloroplast genome (Shinozaki et al., 1986), and its similar expression was also reported in Solanaceae chloroplasts (Legen et al., 2002). However, the validity of *ycf*15 as a protein-coding gene has long been questioned (Steane, 2005; Chumley et al., 2006). Its function was disabled in some basal angiosperms such, as *Amborella* (Goremykin et al., 2003) and *Nuphar* (Raubeson et al., 2007), monocots, most rosids, and some species in other lineages. It has been wholly lost in some other lineages, such as *Illicium*, *Acorus*, *Ceratophyllum*, and *Ranunculus* during their evolution processes. Transcriptome analyses revealed that *ycf*15 has transcribed as a precursor polycistronic transcript that contained *ycf*2, *ycf*15, and antisense *trn*L-CAA. Pseudogene *ycf*15 was mapped by multiple transcripts, which suggested that plastid DNA posttranscriptional splicing might involve a complex cleavage of non-functional genes (Shi et al., 2013).

A similar pseudogenetic elimination also occurred in the LSC of clade III species (*Agropyron*, *Eremopyrum*, *Australopyrum*, and *Henradia*), such as the *rpl*23 pseudogene copy and the *acc*D pseudogene between the *rbc*L gene and the *psa*I gene (Supplementary Figure 2). In the grass plastome, the *rpl*23 gene is originally located in the IR region and encodes the functional ribosomal protein L23. Present studies have indicated that *rpl*23 has been non-reciprocally translocated to a region downstream from *rbc*L (Bowman et al., 1988; Ogihara et al., 1988; He et al., 2017; Lencina et al., 2019). A non-reciprocal translocation of the *rpl*23 gene occurred during the differentiation of *Poaceae* from its unknown ancestor (Katayama and Ogihara, 1996). In this study, we detected only ∼260 bp of *rpl*23 at the 3′ end (complete gene size was 282 bp), which was inserted into the region between *rbc*L and *psa*I within the LSC of Triticeae species (except for *Aegilops tauschii*, *Agropyron*, *Australopyrum*, *Eremopyrum*, *Henradia*, and *S. cereale*). Meanwhile, sequence analysis detected another inserted pseudogene *acc*D in this region, which is situated downstream of *rpl*23. In angiosperms, the *acc*D gene encodes the protein of the acetyl-CoA carboxylase subunit D in the plastome. It is notable that the loss of *accD* is associated with hotspots of rearrangements in each of the families, introducing sensitivity to the herbicides quizalofop and sethoxydim (Konishi and Sasaki, 1994), and causing an alteration of lipid metabolism in the plants. The loss of the *acc*D gene had been found in four lineages of angiosperms, such as grasses (Hiratsuka et al., 1989; Maier et al., 1995; Katayama and Ogihara, 1996), *Campanulaceae* (Cosner et al., 1997), *Geraniaceae* (Palmer et al., 1987; Chumley et al., 2006), and Aroideae (Henriquez et al., 2021). Katayama and Ogihara (1996) considered *acc*D loss prior to the divergence of the Poales. However, previous studies have shown the absence of the *acc*D pseudogene in at least one species of Triticeae (Ogihara et al., 2002), and the presence of the *acc*D pseudogene of up to 349 bp in *Secale* of the Triticeae species (Aagesen et al., 2005). The highly varied pattern of *acc*D pseudogene presence or gene absence in members of the restiid and graminid clades might be due to the pseudogene being carried as the ancestral state throughout most of the divergence of the Poales (Harris et al., 2013). The mechanism for the insertion of variable sizes in this region is uncertain. In this study, the absence of the *acc*D gene was only detected in species of clade III, *A. speltoides*, and *A. tauschii* in Triticeae (Supplementary Figure 3). The *acc*D gene loss might accelerate gene relocations by unknown mechanisms, and various movements of genes might induce the sequential loss of *acc*D (Lee et al., 2007).

The monogenomic Triticeae was diverged about 12–15 MYA to generate lineages, resulting in four branches of plastome species. Our ancestral genome reconstruction suggests that ancestral plastome species of clades II and III lost redundant sequences during divergence from Triticeae. The cp DNA sizes of species in the clades II and III (*Pseudoroegneria*, *Dasypyrum*, *Eremopyrum*, *Lophopyrum*, *Thinopyrum*, *Agropyron*, *Australopyrum*, and *Henradia*) evolved toward size reduction (Figures 3, 6) because of elimination of pseudogene and loss of long fragments (>200 bp) in IGS. Compared with species in clades II and III, the cp genomes of species in clades I and IV (*Aegilops*/*Triticum* complex, *Taeniatherum*, *Secale*, *Crithopsis*, *Herteranthelium*, and *Hordeum*) had larger cp genome size, and retained the invalid genes and lots of redundant fragments of IGS (Figures 3, 6). The cpDNA size reduction in the species of clades II and III (*Pseudoroegneria*, *Dasypyrum*, *Eremopyrum*, *Lophopyrum*, *Thinopyrum, Agropyron*, *Australopyrum*, and *Henradia*) might increase the efficiency of genome replication. Genome reduction is speculated to be the result of a low-cost strategy that could facilitate rapid genome replication under disadvantageous environmental conditions (McCoy et al., 2008; Wu et al., 2009). The retained pseudogenes and replicates might participate in some nucleo-plasmic interactions to promote gene function. Although the *acc*D, *ycf*2, and *ycf*15 genes had been lost from the plastid genome several times in angiosperms, their functions were fulfilled by nuclear copies (Nakkaew et al., 2008; Huang et al., 2017; Wu et al., 2017).

Environmental conditions were thought to influence organelle DNA architecture. For example, plastid genomic compaction in the endolithic ulvophyte seaweed (*Ostreobium quekettii*) and the palmophylalean green alga (*Verdigellas peltata*) was caused primarily by adaptation to low light conditions (Marcelino et al., 2016). The large size variation in major lineages and their subclades is most likely the consequence of adaptive processes since those variations are highly positively correlated with divergence time (Figure 5). Plastid genomes smaller than 120,000 bp were detected mostly in non-photosynthetic angiosperms that had a deletion of several genes during their diversification (Wicke et al., 2011, 2016). The diversification of high plant species has been found to be strongly linked to climate fluctuations (Jaramillo et al., 2006; Hoorn et al., 2010). During the late Miocene (5–10 MYA), the atmospheric CO_2_ level was decreased to the bottom after the mid-Miocene climate optimum (14–16 MYA) (Tripati et al., 2009), resulting in climate change from greenhouse to the icehouse. In order to adapt to an extremely cold and low CO_2_ concentration climate, most species will reduce metabolism to maintain survival, and correspondingly, the expression of genes related to photosynthesis would be reduced, since CO_2_ is required for photosynthesis. The species in the clade I from genera *Aegilops*, *Triticum*, *Secale*, *Taeniatherum*, *Crithopsis*, and *Heteranthelium* were restrictedly distributed in the Mediterranean and adjacent regions (Sakamoto, 1973; Hsiao et al., 1995), where there are hot, dry environments such as the deserts of Mediterranean regions. The sizes of complete cp genome and non-protein coding sequences in Mediterranean lineage were larger than those of other genera as the ancestral species evolved and diverged 0.03–12.71 MYA (Figure 3), and variations appeared most frequently about 5 MYA (Figure 5). The main diversification of Mediterranean lineage in Triticeae occurred about 9 MYA when Mediterranean climates are thought to have arisen. In the late Miocene, when atmospheric CO_2_ concentrations and temperatures were extremely low, the Mediterranean climate undoubtedly provided a good shelter for some plants. The development of the Mediterranean climate can be seen as the opening of a new and novel climatic niche, to which lineages have adapted and speciated, by accumulating morphological change in other climate zones (Yesson and Culham, 2006). It is, thus, likely that climate oscillations during the late Miocene, especially the establishment of the Mediterranean climate, might have promoted the Mediterranean lineage of Triticeae rapid diversification and adaptation, and have continued to diversify from the Quaternary to the present (Fan et al., 2013), which resulted in plastid genome and nuclear genome change to adapt to climate oscillations. We suggested that the dynamically reduced/enlarged cpDNAs of Triticeae might result from the adaptation to historical climate changes. However, we still need more molecular evidence to determine the role of natural selection in chloroplast genome evolution during the diversification of Triticeae.

## Conclusion

In this study, we detected gene loss/pseudonization, indels, and intron variation, variation in cp genome sequence size, and expansion/contraction in IRs among 34 chloroplast genomes of monogenomic Triticeae species. We found that the cp genome sequence size variation was mainly caused by the size of non-protein coding sequences. The monogenomic Triticeae diverged about 12–15 MYA, which resulted in four stem branches of plastome species. Losses of a series of invalid genes or sequence fragments have no effect on genomic function in these plants, which might be an evolutionary mechanism to increase the efficiency of genome replication. According to the distribution and habitat of the species, the species in the Mediterranean region (in clade I, *Aegilops*/*Triticum* complex, *Taeniatherum*, *Secale*, *Crithopsis*, and *Herteranthelium*) might have experienced the change in the Mediterranean climate and expanded the cp genome significantly. Our results enhance the understanding of the complexity and evolution of Triticeae cp DNAs.

## Data Availability Statement

Publicly available datasets were analyzed in this study. This data can be found here: the chloroplast genome sequences in this study, KJ614418, KJ614416, KY636033, KJ614413, KJ614419, KJ614406, KJ614405, KJ614412, KY636059, KY636056, KY126307, MH285848, KY636075, MH331642, MH285849, MH285850, MH285851, MH285852, MH285853, MH285854, MH331641, KM974741, EF115541, KC912689, MH331643, MH331640, KX822019, MH285855, KC912691, MH285856, MH331639, LC005977, KC912692, KJ614411, and EU325680 are available in NCBI.

## Author Contributions

NC and XF designed the experiments. NC carried out the experiments, analyzed the experimental results and data, and developed the analysis tools. L-NS, Y-LW, L-JY, YZ, YW, D-DW, H-YK, H-QZ, Y-HZ, and G-LS assisted in writing the manuscript. All authors contributed to the article and approved the submitted version.

## Conflict of Interest

The authors declare that the research was conducted in the absence of any commercial or financial relationships that could be construed as a potential conflict of interest.

## Publisher’s Note

All claims expressed in this article are solely those of the authors and do not necessarily represent those of their affiliated organizations, or those of the publisher, the editors and the reviewers. Any product that may be evaluated in this article, or claim that may be made by its manufacturer, is not guaranteed or endorsed by the publisher.

## Footnotes

1
http://tree.bio.ed.ac.uk/

## Supplementary Material

Supplementary Figure 1 Comparison of the chloroplast genome. Alignment of the cp genome sequences of 34 Triticeae species and *Brachypodium distachyon* generated with mVISTA. Gray arrows indicate the position and direction of each gene. Red and blue areas indicate intergenic and genic regions, respectively.

Supplementary Figures 2–17 Indels in protein coding genes, gene loss/pseudonization, intron variation, and intergenic sequence (IGS) of 34 Triticeae diploids cp genomes.

## Abbreviations

Cp: chloroplast
Ir: inverted repeat
Lsc: large-single-copy
Ssc: small single copy
Igs: intergenic region sequence
Ml: maximum likelihood
Bi: Bayesian inference
Mcmc: Markov Chain Monte Carlo
Bs: bootstrapping.
