# Supplementary figures and images for: Variation in Plastome Sizes Accompanied by Evolutionary History in Monogenomic Triticeae (Poaceae: Triticeae)
Source: Front Plant Sci. 2021 Dec 13;12:741063. doi: 10.3389/fpls.2021.741063 (PMC8710740; doi:10.3389/fpls.2021.741063)

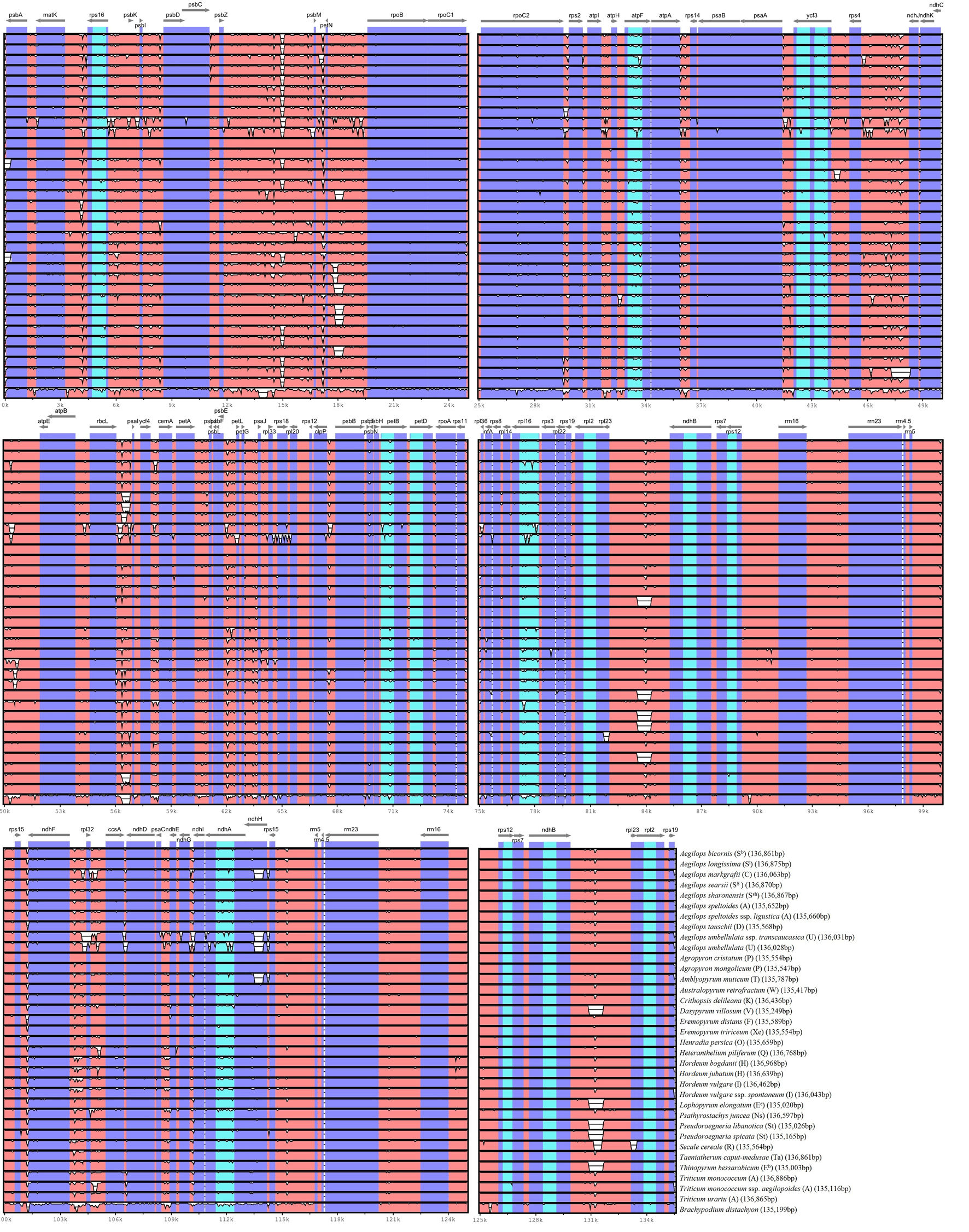

Supplement: Supplementary Figure 1 — Comparison of the chloroplast genome. Alignment of the cp genome sequences of 34 Triticeae species and Brachypodium distachyon generated with mVISTA. Gray arrows indicate the position and direction of each gene. Red and blue areas indicate intergenic and genic regions, respectively. [file Image_1.JPEG]
