# Supplementary material for: Variation in Plastome Sizes Accompanied by Evolutionary History in Monogenomic Triticeae (Poaceae: Triticeae)
Source: Front Plant Sci. 2021 Dec 13;12:741063. doi: 10.3389/fpls.2021.741063 (PMC8710740; doi:10.3389/fpls.2021.741063)
Supplement: Supplementary Figures 2–17 — Indels in protein coding genes, gene loss/pseudonization, intron variation, and intergenic sequence (IGS) of 34 Triticeae diploids cp genomes. [file Data_Sheet_1.PDF]

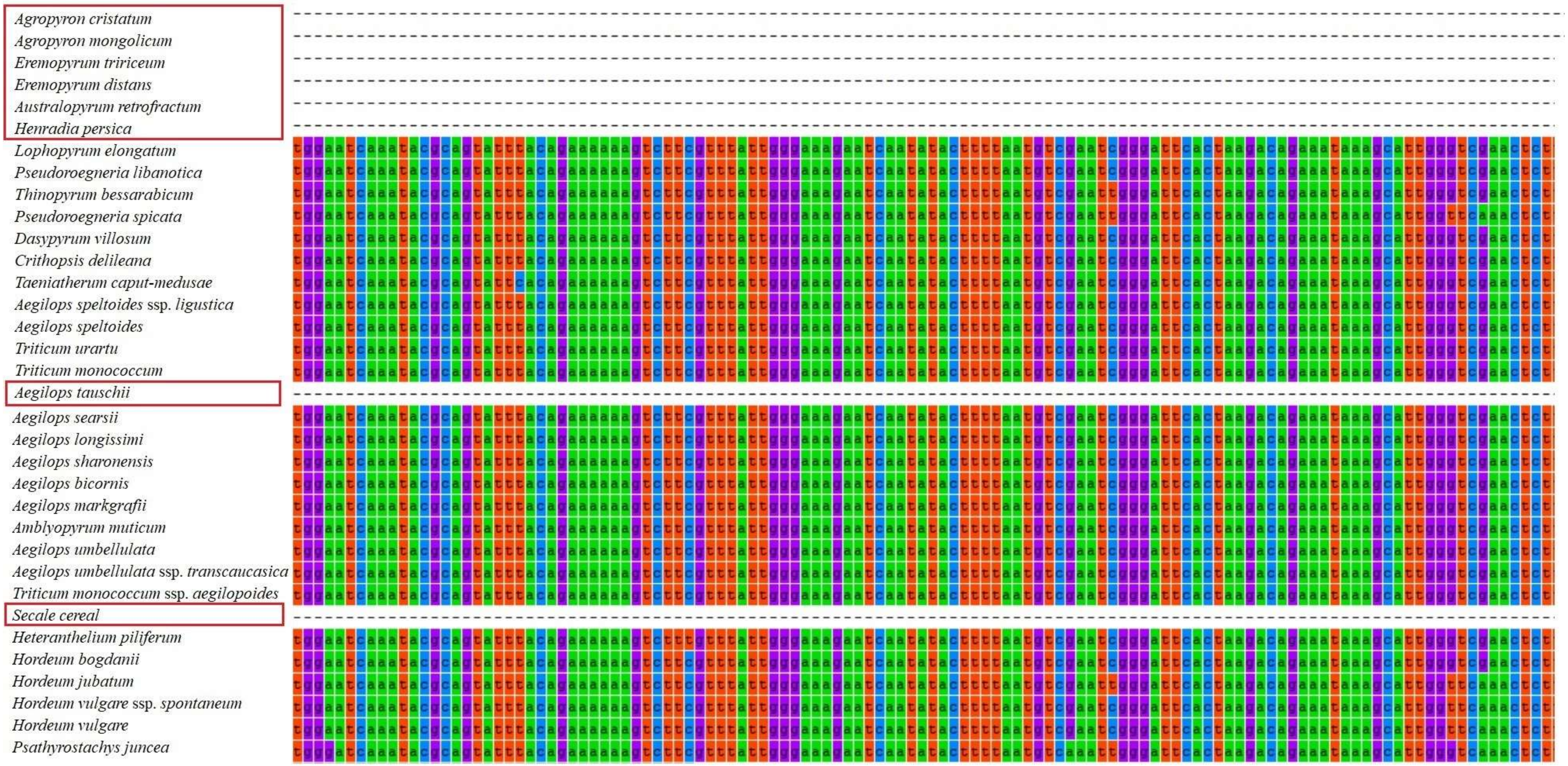
Agropyron cristatum
Agropyron mongolicum
Eremopyrum tririceum
Eremopyrum distans
Australopyrum retrofractum
Henradia persica
Lophopyrum elongatum
Pseudoroegneria libanotica
Thinopyrum bessarabicum
Pseudoroegneria spicata
Dasypyrum villosum
Crithopsis delileana
Taeniatherum caput-medusae
Aegilops speltoides ssp. ligustica
Aegilops speltoides
Triticum urartu
Triticum monococcum
Aegilops tauschii
Aegilops searsii
Aegilops longissimi
Aegilops sharonensis
Aegilops bicornis
Aegilops markgrafii
Amblyopyrum muticum
Aegilops umbellulata
Aegilops umbellulata ssp. transcaucasica
Triticum monococcum ssp. aegilopoides
Secale cereal
Heteranthelium piliferum
Hordeum bogdanii
Hordeum jubatum
Hordeum vulgare ssp. spontaneum
Hordeum vulgare
Psathyrostachys juncea

S2 *rbc*L-*psa*I

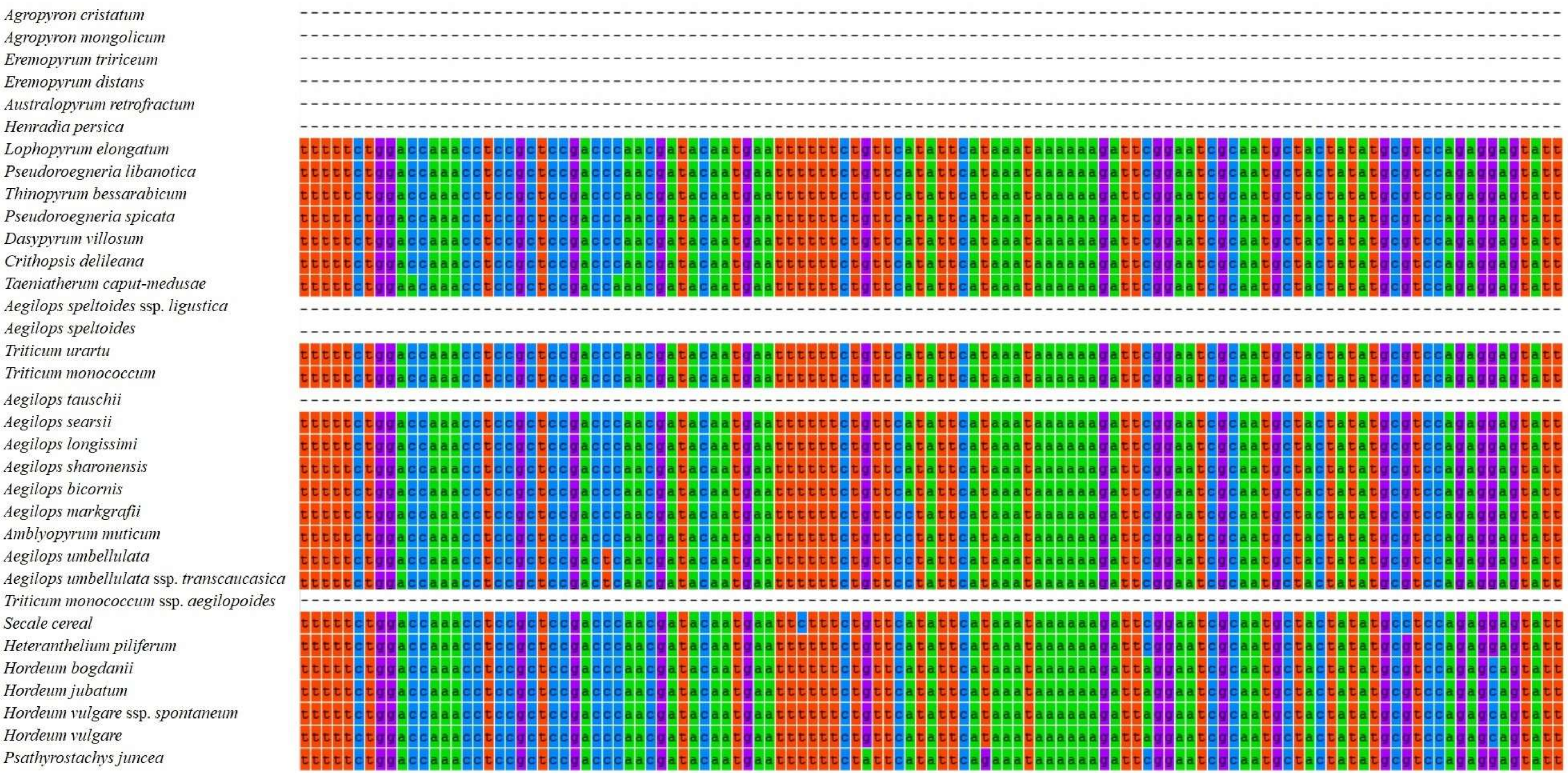
Agropyron cristatum
Agropyron mongolicum
Eremopyrum tririceum
Eremopyrum distans
Australopyrum retrofractum
Henradia persica
Lophopyrum elongatum
Pseudoroegneria libanotica
Thinopyrum bessarabicum
Pseudoroegneria spicata
Dasypyrum villosum
Crithopsis delileana
Taeniatherum caput-medusae
Aegilops speltoides ssp. ligustica
Aegilops speltoides
Triticum urartu
Triticum monococcum
Aegilops tauschii
Aegilops searsii
Aegilops longissimi
Aegilops sharonensis
Aegilops bicornis
Aegilops markgrafii
Amblyopyrum muticum
Aegilops umbellulata
Aegilops umbellulata ssp. transcaucasica
Triticum monococcum ssp. aegilopoides
Secale cereal
Heteranthelium piliferum
Hordeum bogdanii
Hordeum jubatum
Hordeum vulgare ssp. spontaneum
Hordeum vulgare
Psathyrostachys juncea

S3 *acc*D

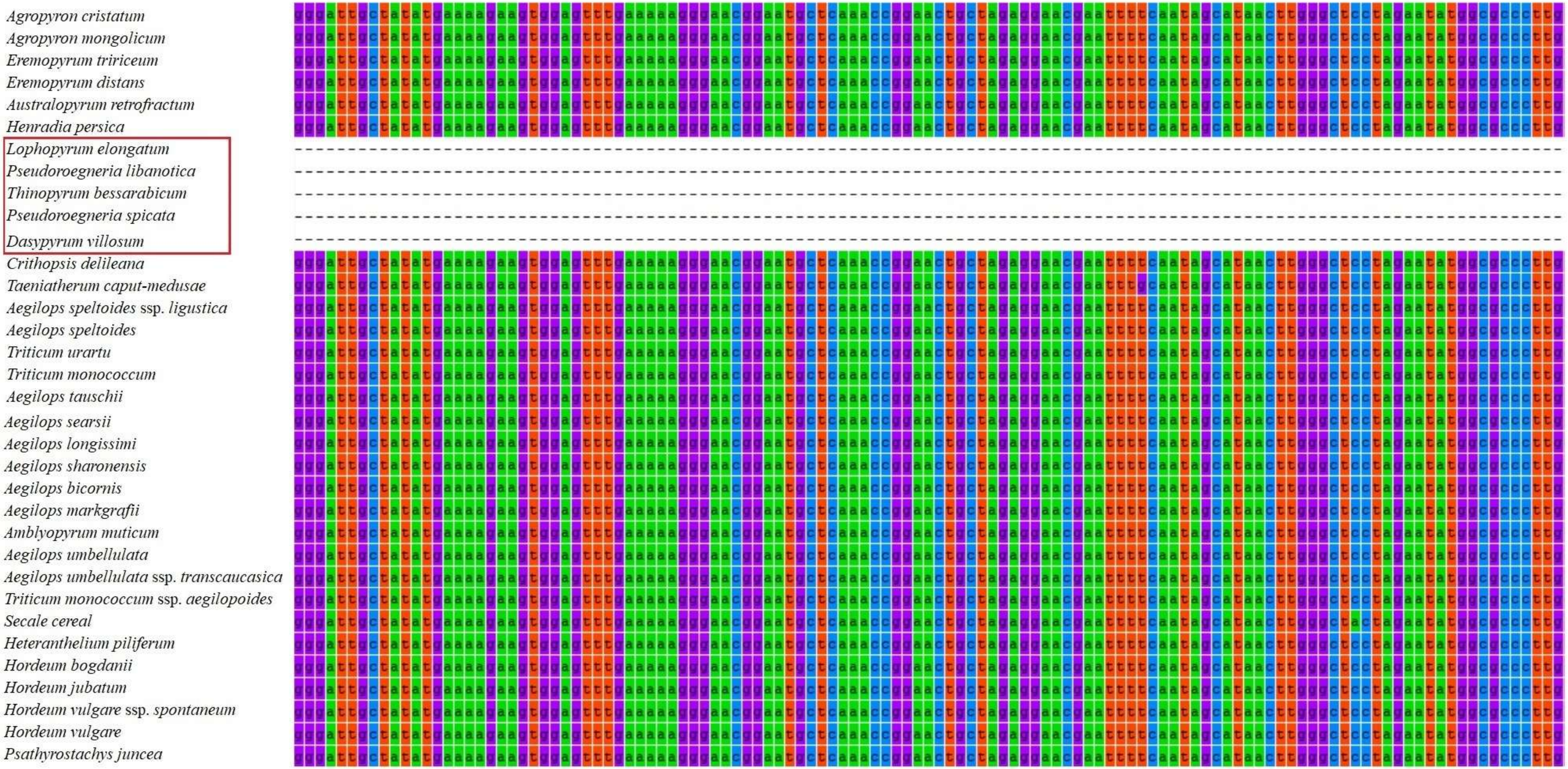
Agropyron cristatum
Agropyron mongolicum
Eremopyrum tririceum
Eremopyrum distans
Australopyrum retrofractum
Henradia persica
Lophopyrum elongatum
Pseudoroegneria libanotica
Thinopyrum bessarabicum
Pseudoroegneria spicata
Dasypyrum villosum
Crithopsis delileana
Taeniatherum caput-medusae
Aegilops speltoides ssp. ligustica
Aegilops speltoides
Triticum urartu
Triticum monococcum
Aegilops tauschii
Aegilops searsii
Aegilops longissimi
Aegilops sharonensis
Aegilops bicornis
Aegilops markgrafii
Amblyopyrum muticum
Aegilops umbellulata
Aegilops umbellulata ssp. transcaucasica
Triticum monococcum ssp. aegilopoides
Secale cereal
Heteranthelium piliferum
Hordeum bogdanii
Hordeum jubatum
Hordeum vulgare ssp. spontaneum
Hordeum vulgare
Psathyrostachys juncea

S4 *trn*L-*trn*I

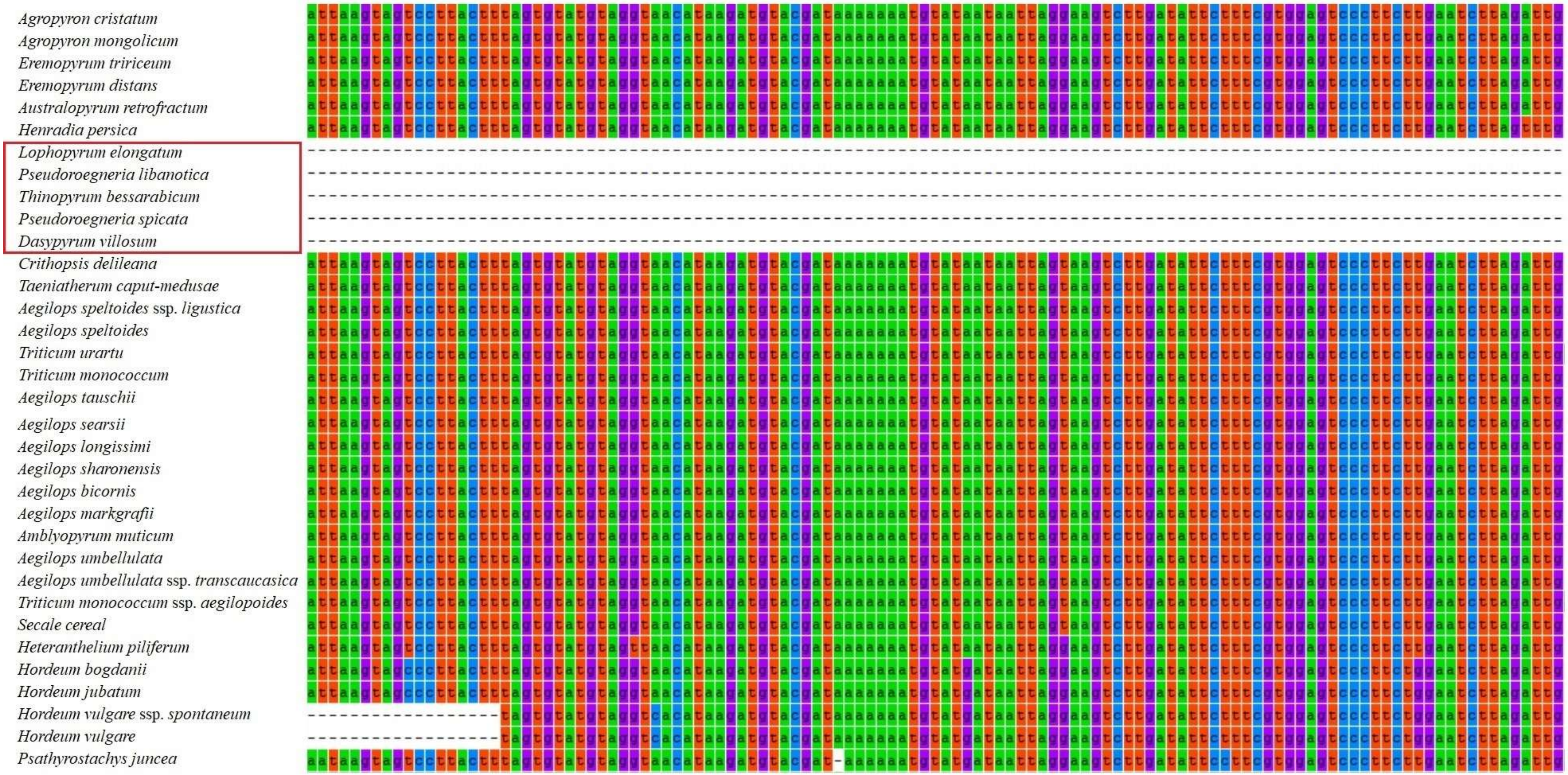
Agropyron cristatum
Agropyron mongolicum
Eremopyrum tririceum
Eremopyrum distans
Australopyrum retrofractum
Henradia persica
Lophopyrum elongatum
Pseudoroegneria libanotica
Thinopyrum bessarabicum
Pseudoroegneria spicata
Dasypyrum villosum
Crithopsis delileana
Taeniatherum caput-medusae
Aegilops speltoides ssp. ligustica
Aegilops speltoides
Triticum urartu
Triticum monococcum
Aegilops tauschii
Aegilops searsii
Aegilops longissimi
Aegilops sharonensis
Aegilops bicornis
Aegilops markgrafii
Amblyopyrum muticum
Aegilops umbellulata
Aegilops umbellulata ssp. transcaucasica
Triticum monococcum ssp. aegilopoides
Secale cereal
Heteranthelium piliferum
Hordeum bogdanii
Hordeum jubatum
Hordeum vulgare ssp. spontaneum
Hordeum vulgare
Psathyrostachys juncea

S5 *pet*N-*trn*C

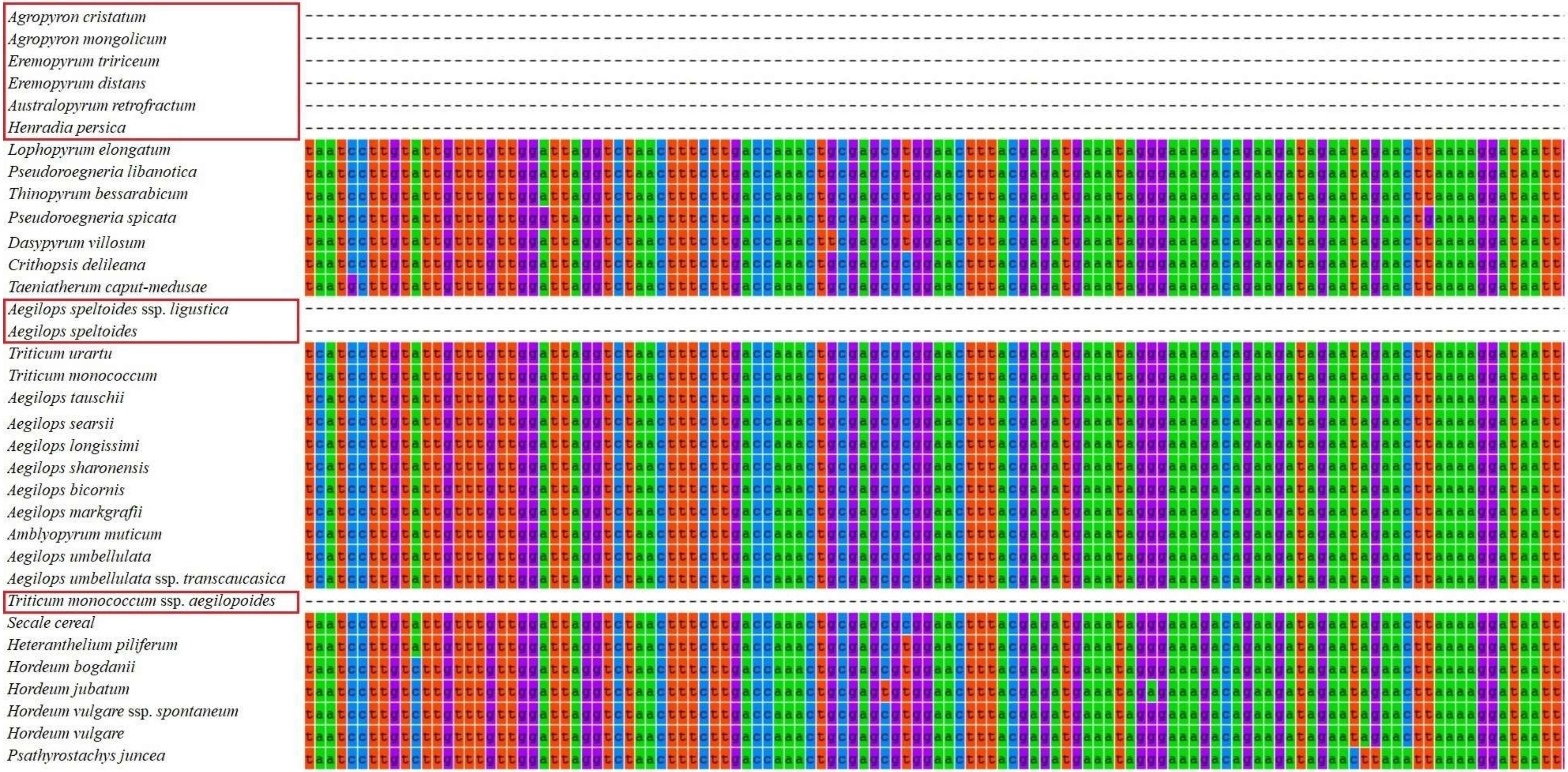
Agropyron cristatum
Agropyron mongolicum
Eremopyrum tririceum
Eremopyrum distans
Australopyrum retrofractum
Henradia persica
Lophopyrum elongatum
Pseudoroegneria libanotica
Thinopyrum bessarabicum
Pseudoroegneria spicata
Dasypyrum villosum
Crithopsis delileana
Taeniatherum caput-medusae
Aegilops speltoides ssp. ligustica
Aegilops speltoides
Triticum urartu
Triticum monococcum
Aegilops tauschii
Aegilops searsii
Aegilops longissimi
Aegilops sharonensis
Aegilops bicornis
Aegilops markgrafii
Amblyopyrum muticum
Aegilops umbellulata
Aegilops umbellulata ssp. transcaucasica
Triticum monococcum ssp. aegilopoides
Secale cereal
Heteranthelium piliferum
Hordeum bogdanii
Hordeum jubatum
Hordeum vulgare ssp. spontaneum
Hordeum vulgare
Psathyrostachys juncea

S6 *psb*E-*pet*L

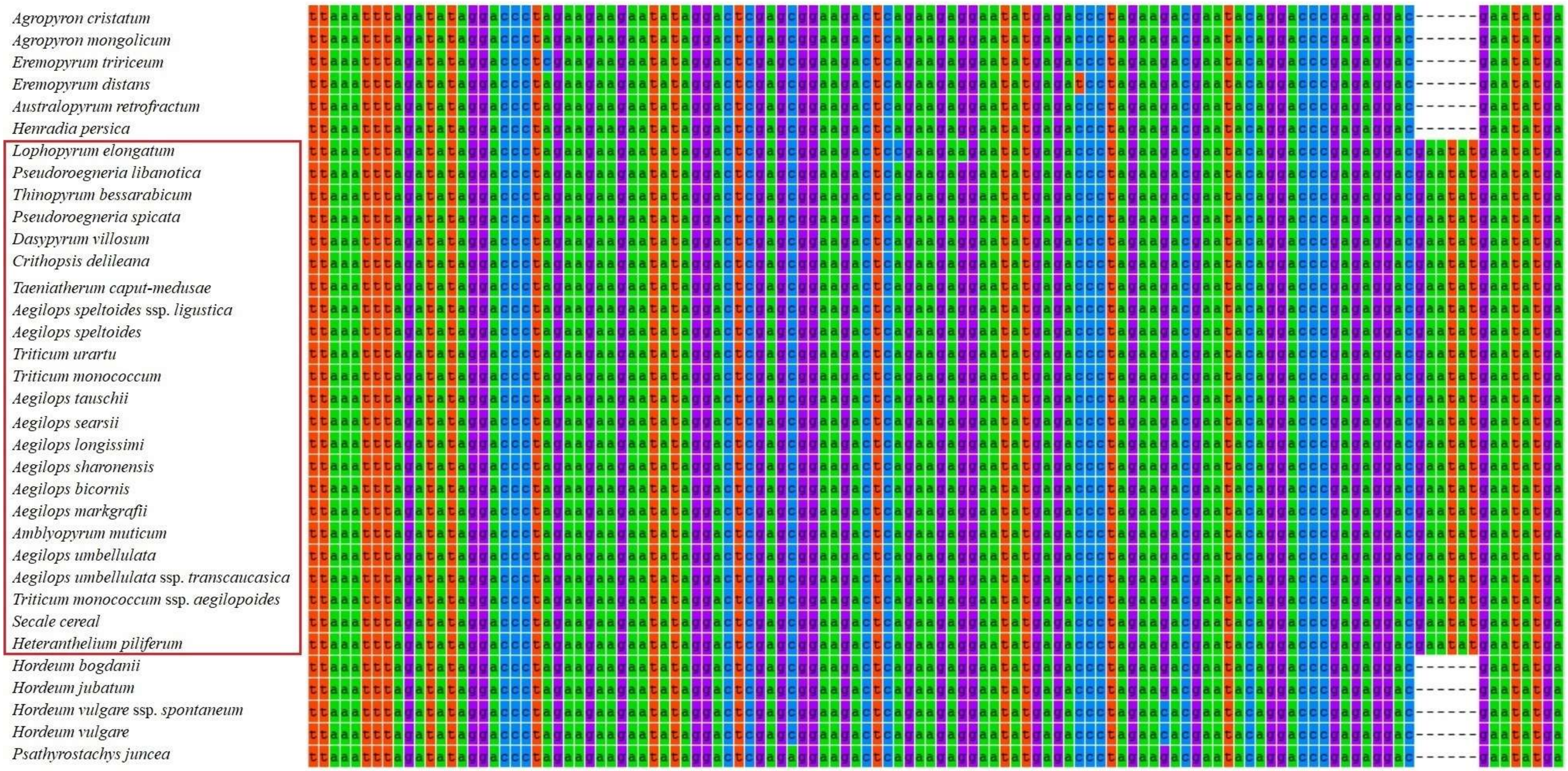
Agropyron cristatum
Agropyron mongolicum
Eremopyrum tririceum
Eremopyrum distans
Australopyrum retrofractum
Henradia persica
Lophopyrum elongatum
Pseudoroegneria libanotica
Thinopyrum bessarabicum
Pseudoroegneria spicata
Dasypyrum villosum
Crithopsis delileana
Taeniatherum caput-medusae
Aegilops speltoides ssp. ligustica
Aegilops speltoides
Triticum urartu
Triticum monococcum
Aegilops tauschii
Aegilops searsii
Aegilops longissimi
Aegilops sharonensis
Aegilops bicornis
Aegilops markgrafii
Amblyopyrum muticum
Aegilops umbellulata
Aegilops umbellulata ssp. transcaucasica
Triticum monococcum ssp. aegilopoides
Secale cereal
Heteranthelium piliferum
Hordeum bogdanii
Hordeum jubatum
Hordeum vulgare ssp. spontaneum
Hordeum vulgare
Psathyrostachys juncea

S7 *rpo*C2-1

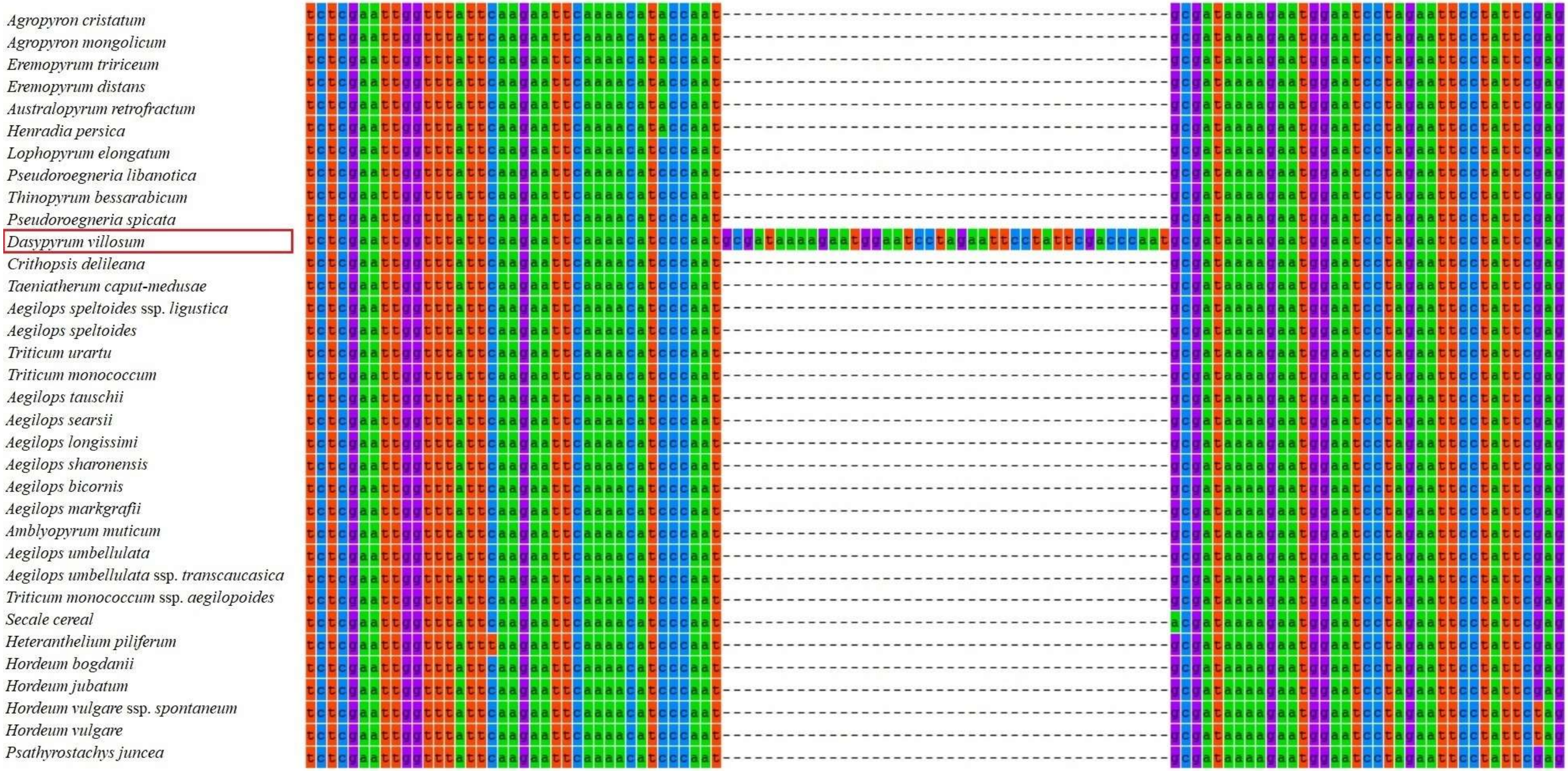

Agropyron cristatum
Agropyron mongolicum
Eremopyrum tririceum
Eremopyrum distans
Australopyrum retrofractum
Henradia persica
Lophopyrum elongatum
Pseudoroegneria libanotica
Thinopyrum bessarabicum
Pseudoroegneria spicata
Dasypyrum villosum
Crithopsis delileana
Taeniatherum caput-medusae
Aegilops speltoides ssp. ligustica
Aegilops speltoides
Triticum urartu
Triticum monococcum
Aegilops tauschii
Aegilops searsii
Aegilops longissimi
Aegilops sharonensis
Aegilops bicornis
Aegilops markgrafii
Amblyopyrum muticum
Aegilops umbellulata
Aegilops umbellulata ssp. transcaucasica
Triticum monococcum ssp. aegilopoides
Secale cereal
Heteranthelium piliferum
Hordeum bogdanii
Hordeum jubatum
Hordeum vulgare ssp. spontaneum
Hordeum vulgare
Psathyrostachys juncea


S8 *rpo*C2

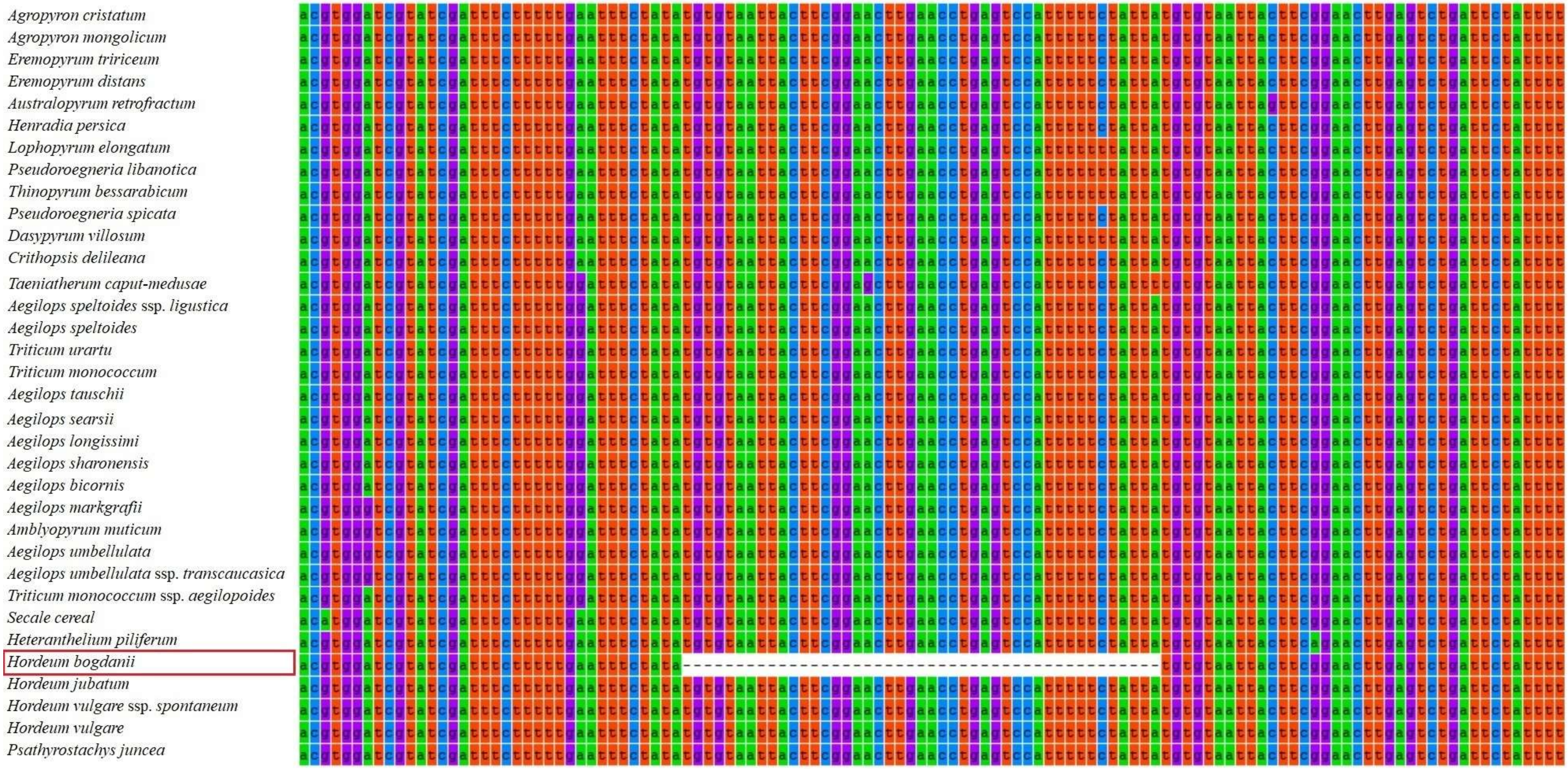
*Agropyron cristatum*
*Agropyron mongolicum*
*Eremopyrum triticeum*
*Eremopyrum distans*
*Australopyrum retrofractum*
*Henradia persica*
*Lophopyrum elongatum*
*Pseudoroegneria libanotica*
*Thinopyrum bessarabicum*
*Pseudoroegneria spicata*
*Dasypyrum villosum*
*Crithopsis delileana*
*Taeniatherum caput-medusae*
*Aegilops speltoides* ssp. *ligustica*
*Aegilops speltoides*
*Triticum urartu*
*Triticum monococcum*
*Aegilops tauschii*
*Aegilops searsii*
*Aegilops longissimi*
*Aegilops sharonensis*
*Aegilops bicornis*
*Aegilops markgrafii*
*Amblyopyrum muticum*
*Aegilops umbellulata*
*Aegilops umbellulata* ssp. *transcaucasica*
*Triticum monococcum* ssp. *aegilopoides*
*Secale cereal*
*Heteranthelium piliferum*
*Hordeum bogdanii*
*Hordeum jubatum*
*Hordeum vulgare* ssp. *spontaneum*
*Hordeum vulgare*
*Psathyrostachys juncea*

S9 *rps3*

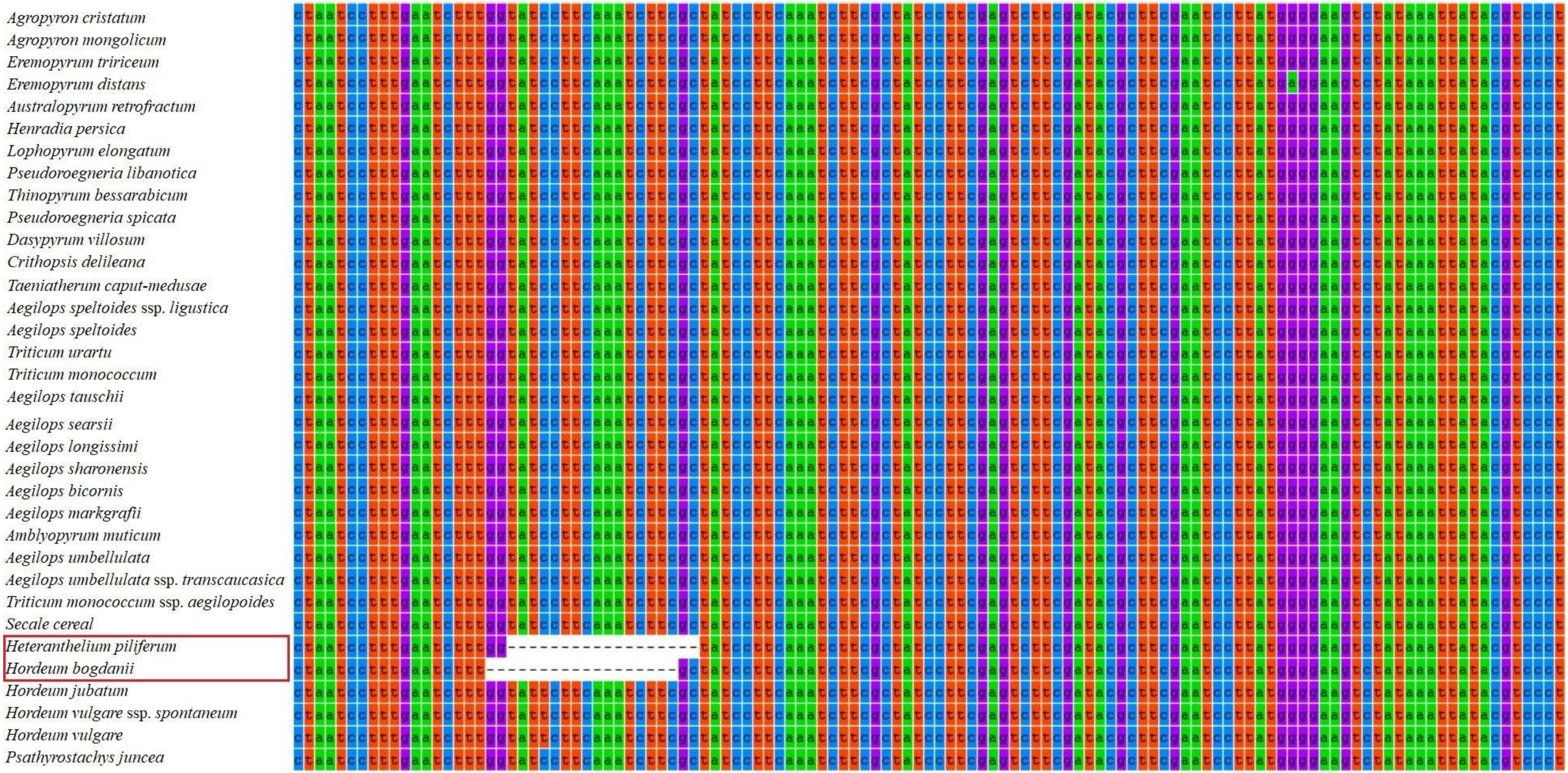
Agropyron cristatum
Agropyron mongolicum
Eremopyrum tririceum
Eremopyrum distans
Australopyrum retrofractum
Henradia persica
Lophopyrum elongatum
Pseudoroegneria libanotica
Thinopyrum bessarabicum
Pseudoroegneria spicata
Dasypyrum villosum
Crithopsis delileana
Taeniatherum caput-medusae
Aegilops speltoides ssp. ligustica
Aegilops speltoides
Triticum urartu
Triticum monococcum
Aegilops tauschii
Aegilops searsii
Aegilops longissimi
Aegilops sharonensis
Aegilops bicornis
Aegilops markgrafii
Amblyopyrum muticum
Aegilops umbellulata
Aegilops umbellulata ssp. transcaucasica
Triticum monococcum ssp. aegilopoides
Secale cereal
Heteranthelium piliferum
Hordeum bogdanii
Hordeum jubatum
Hordeum vulgare ssp. spontaneum
Hordeum vulgare
Psathyrostachys juncea

S10 *inf*A

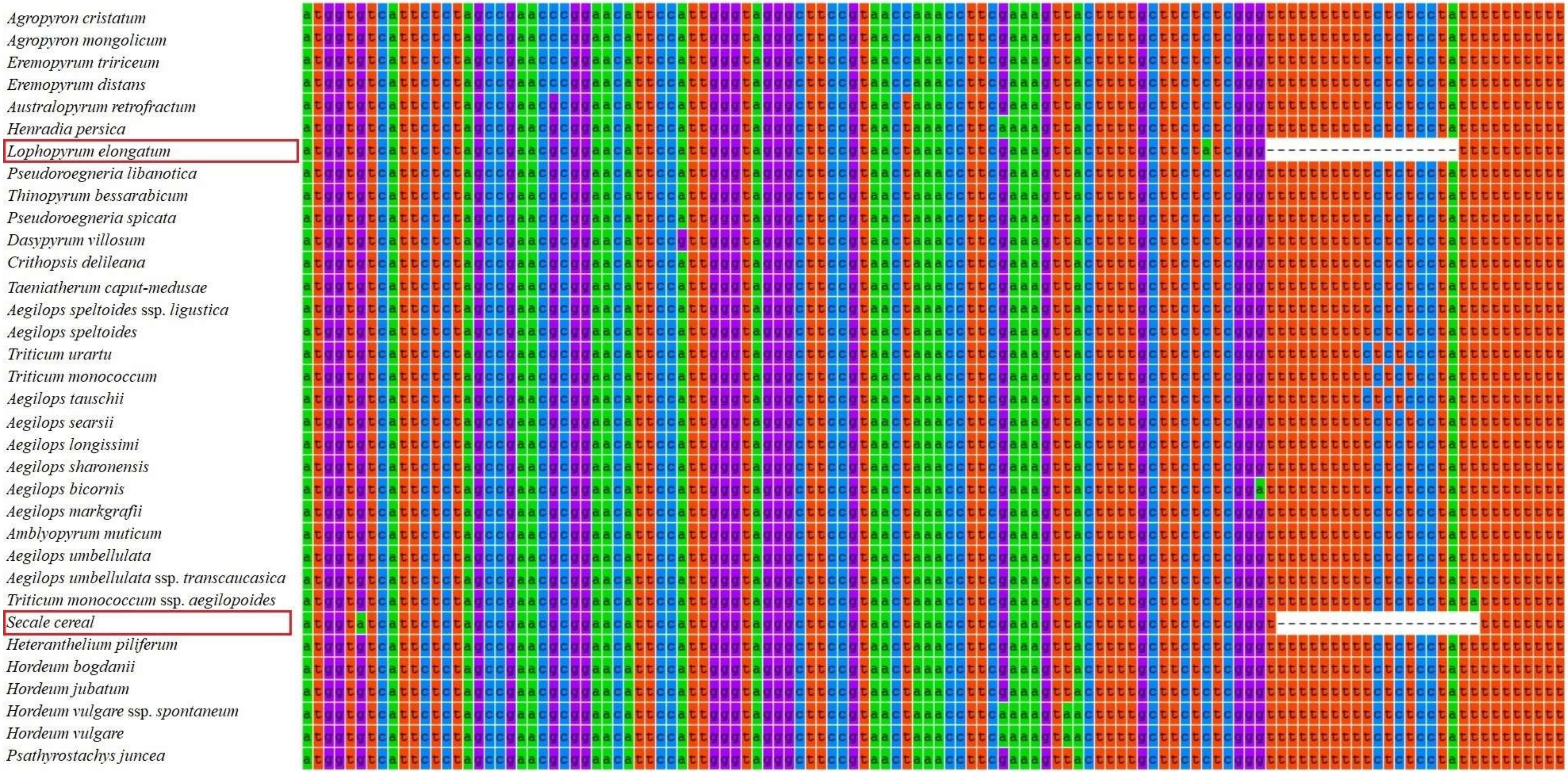
Agropyron cristatum
Agropyron mongolicum
Eremopyrum tririceum
Eremopyrum distans
Australopyrum retrofractum
Henradia persica
Lophopyrum elongatum
Pseudoroegneria libanotica
Thinopyrum bessarabicum
Pseudoroegneria spicata
Dasypyrum villosum
Crithopsis delileana
Taeniatherum caput-medusae
Aegilops speltoides ssp. ligustica
Aegilops speltoides
Triticum urartu
Triticum monococcum
Aegilops tauschii
Aegilops searsii
Aegilops longissimi
Aegilops sharonensis
Aegilops bicornis
Aegilops markgrafii
Amblyopyrum muticum
Aegilops umbellulata
Aegilops umbellulata ssp. transcaucasica
Triticum monococcum ssp. aegilopoides
Secale cereal
Heteranthelium piliferum
Hordeum bogdanii
Hordeum jubatum
Hordeum vulgare ssp. spontaneum
Hordeum vulgare
Psathyrostachys juncea

S11 *inf*A-1

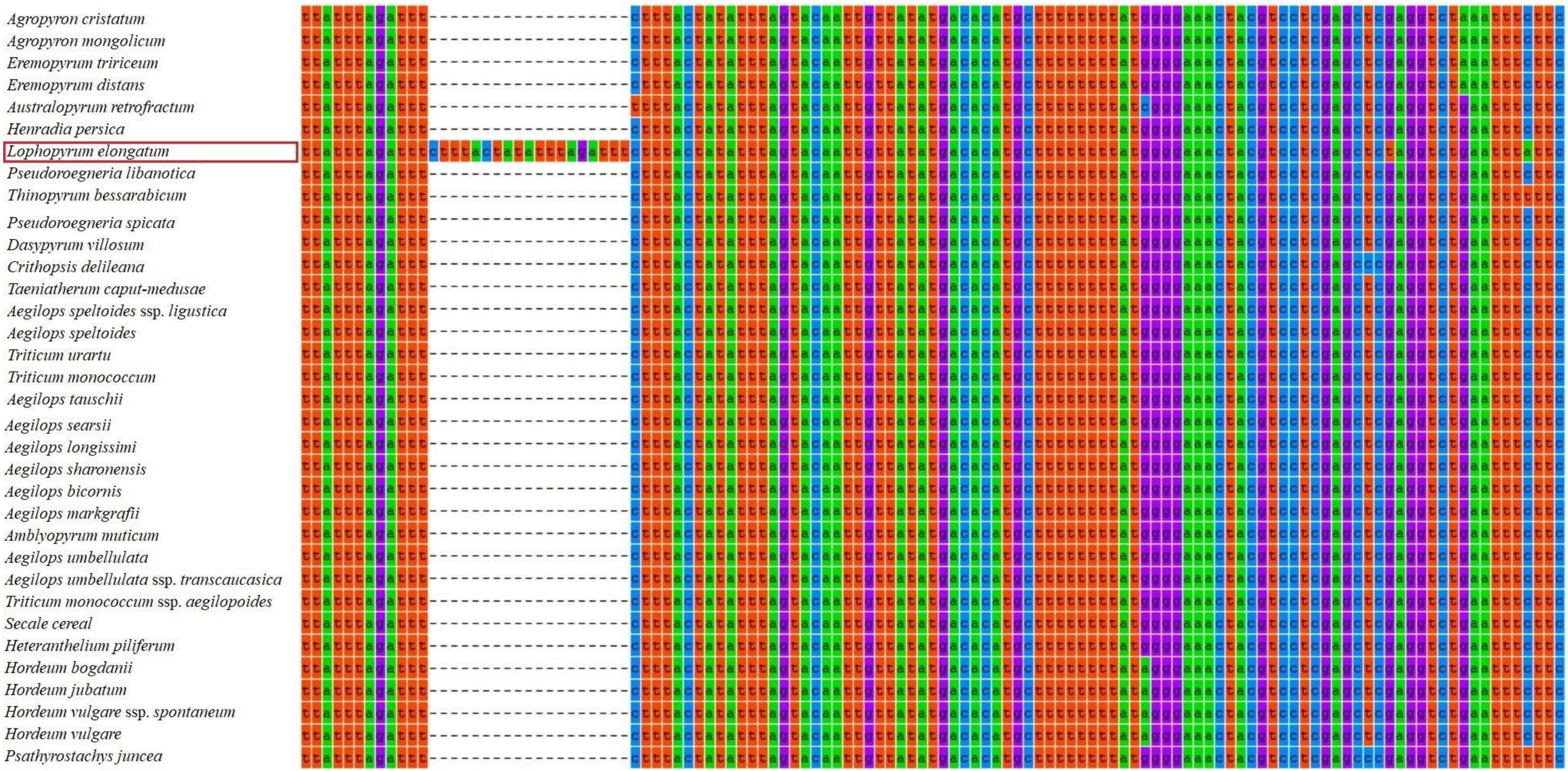
Agropyron cristatum
Agropyron mongolicum
Eremopyrum tririceum
Eremopyrum distans
Australopyrum retrofractum
Henradia persica
Lophopyrum elongatum
Pseudoroegneria libanotica
Thinopyrum bessarabicum
Pseudoroegneria spicata
Dasypyrum villosum
Crithopsis delileana
Taeniatherum caput-medusae
Aegilops speltoides ssp. ligustica
Aegilops speltoides
Triticum urartu
Triticum monococcum
Aegilops tauschii
Aegilops searsii
Aegilops longissimi
Aegilops sharonensis
Aegilops bicornis
Aegilops markgrafii
Amblyopyrum muticum
Aegilops umbellulata
Aegilops umbellulata ssp. transcaucasica
Triticum monococcum ssp. aegilopoides
Secale cereal
Heteranthelium piliferum
Hordeum bogdanii
Hordeum jubatum
Hordeum vulgare ssp. spontaneum
Hordeum vulgare
Psathyrostachys juncea

S12 *rpl*22

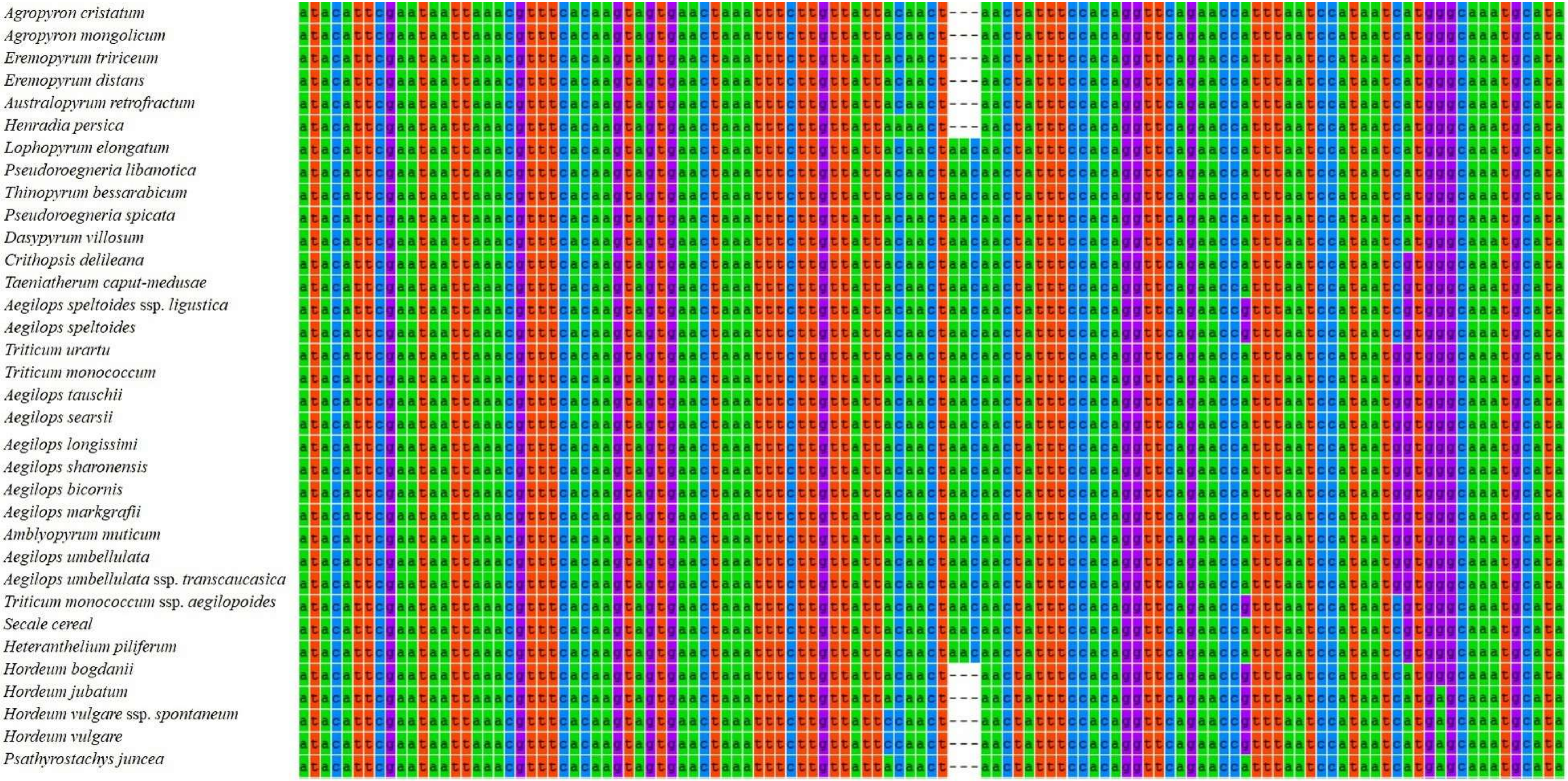
Agropyron cristatum
Agropyron mongolicum
Eremopyrum tririceum
Eremopyrum distans
Australopyrum retrofractum
Henradia persica
Lophopyrum elongatum
Pseudoroegneria libanotica
Thinopyrum bessarabicum
Pseudoroegneria spicata
Dasypyrum villosum
Crithopsis delileana
Taeniatherum caput-medusae
Aegilops speltoides ssp. ligustica
Aegilops speltoides
Triticum urartu
Triticum monococcum
Aegilops tauschii
Aegilops searsii
Aegilops longissimi
Aegilops sharonensis
Aegilops bicornis
Aegilops markgrafii
Amblyopyrum muticum
Aegilops umbellulata
Aegilops umbellulata ssp. transcaucasica
Triticum monococcum ssp. aegilopoides
Secale cereal
Heteranthelium piliferum
Hordeum bogdanii
Hordeum jubatum
Hordeum vulgare ssp. spontaneum
Hordeum vulgare
Psathyrostachys juncea

S13 *mat*K

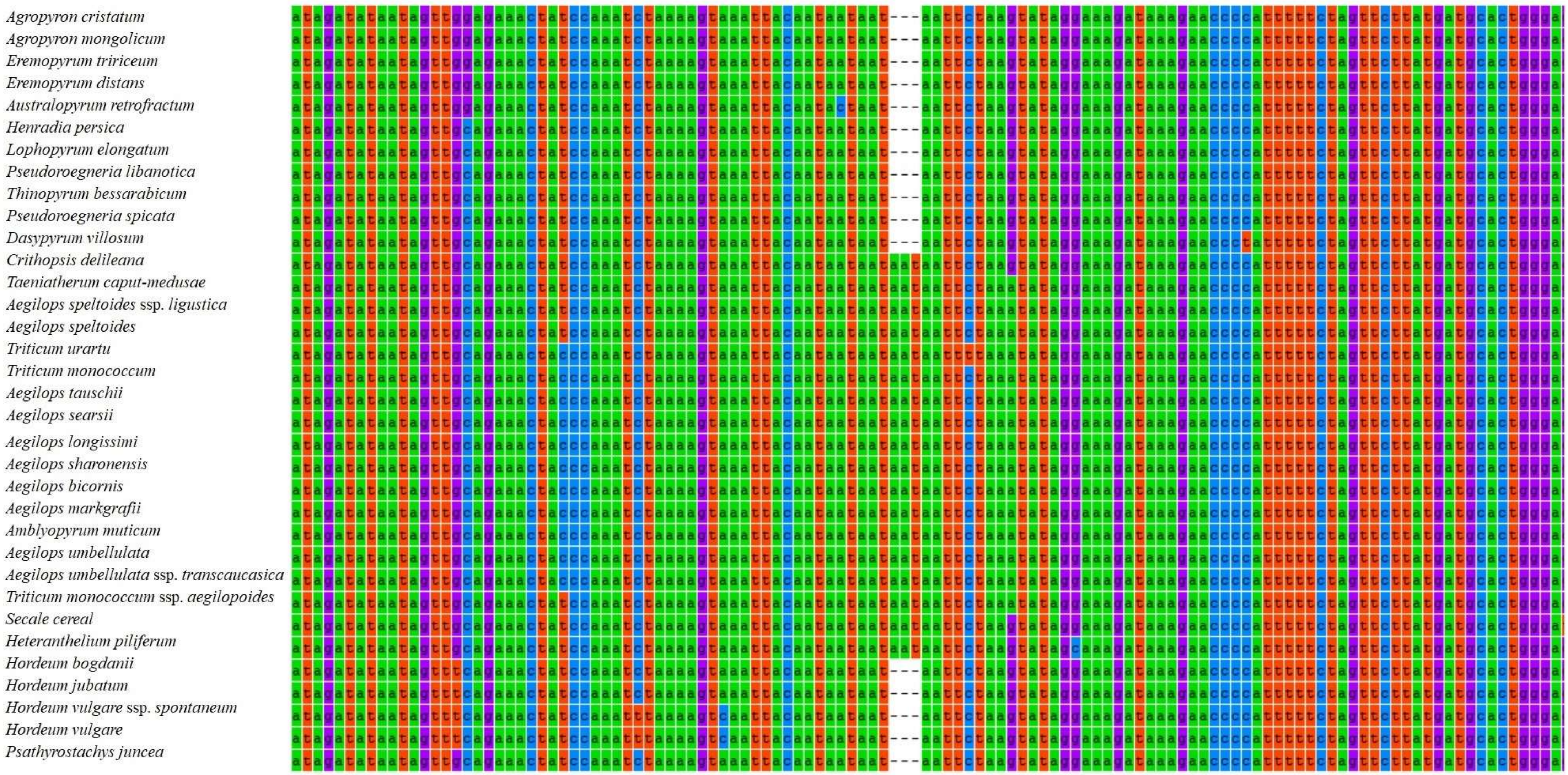
Agropyron cristatum
Agropyron mongolicum
Eremopyrum tririceum
Eremopyrum distans
Australopyrum retrofractum
Henradia persica
Lophopyrum elongatum
Pseudoroegneria libanotica
Thinopyrum bessarabicum
Pseudoroegneria spicata
Dasypyrum villosum
Crithopsis delileana
Taeniatherum caput-medusae
Aegilops speltoides ssp. ligustica
Aegilops speltoides
Triticum urartu
Triticum monococcum
Aegilops tauschii
Aegilops searsii
Aegilops longissimi
Aegilops sharonensis
Aegilops bicornis
Aegilops markgrafii
Amblyopyrum muticum
Aegilops umbellulata
Aegilops umbellulata ssp. transcaucasica
Triticum monococcum ssp. aegilopoides
Secale cereal
Heteranthelium piliferum
Hordeum bogdanii
Hordeum jubatum
Hordeum vulgare ssp. spontaneum
Hordeum vulgare
Psathyrostachys juncea

S14 *rpo*C1

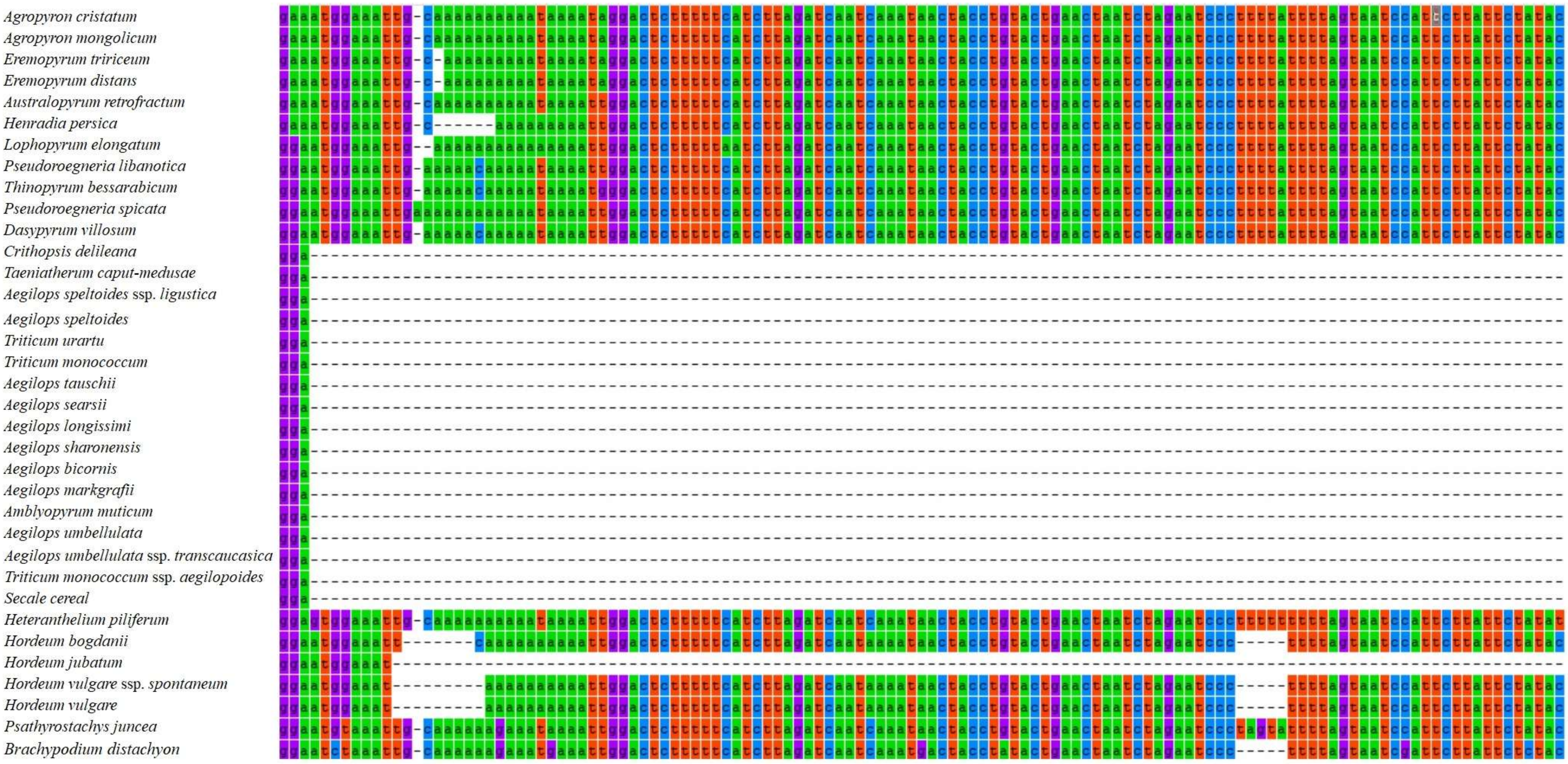


S15 *trn*T~*trn*E

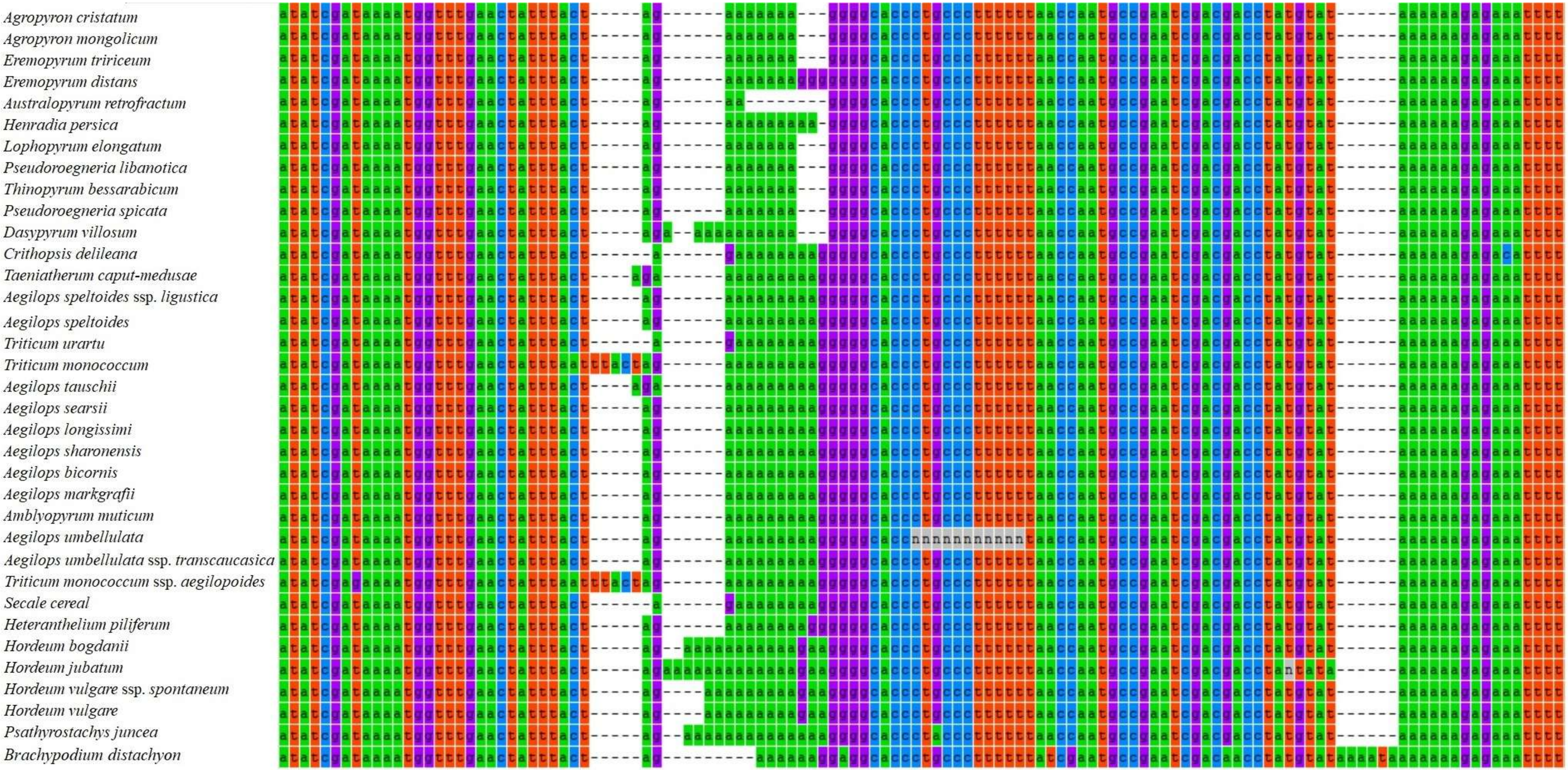


S16 *atp*F intron

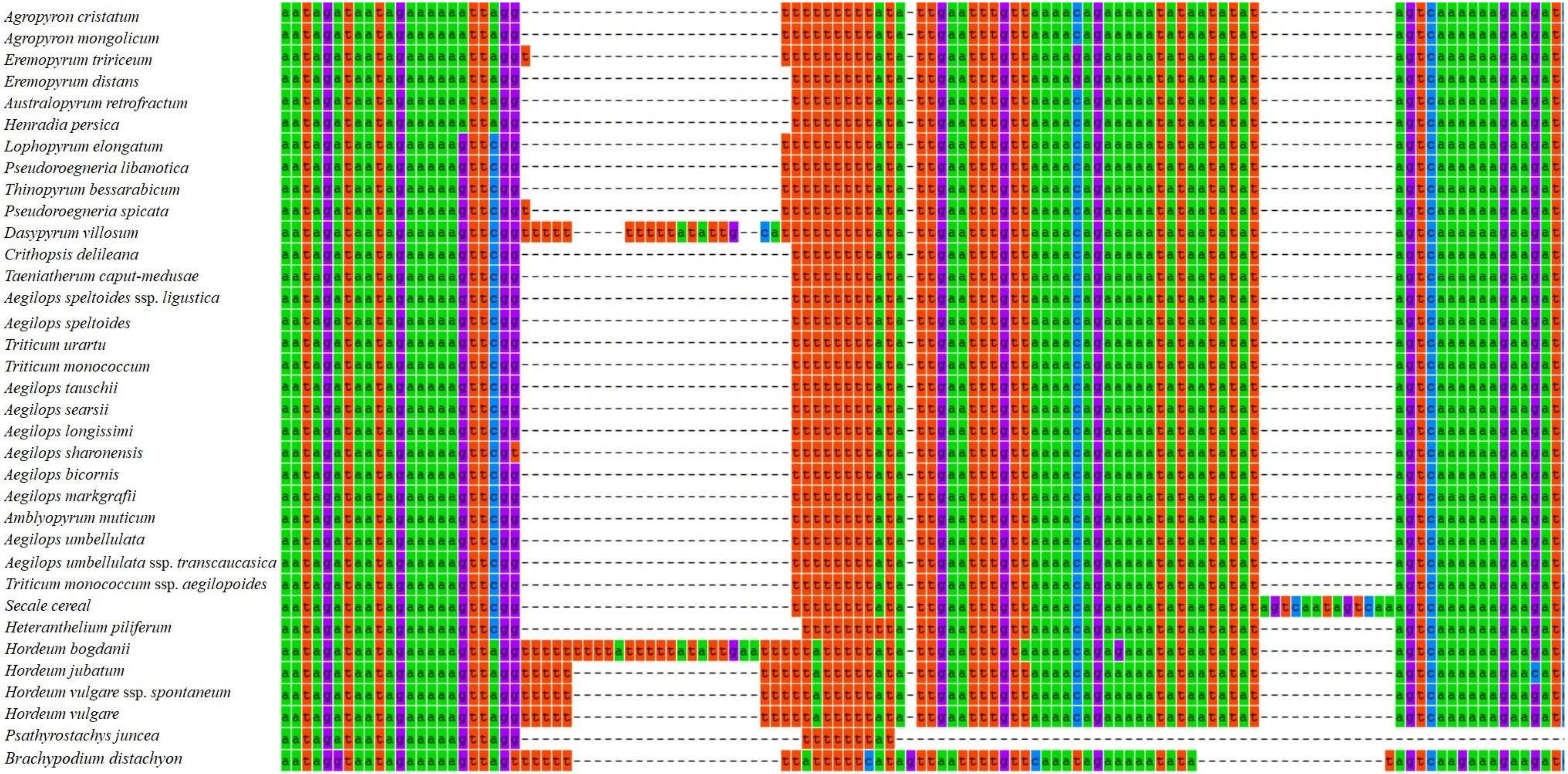


S17 *rpl*16 intron
